# The two-component system CrdRS mediates sub-inhibitory antibiotic-induced biofilm formation in *Helicobacter pylori* via bidirectional regulation of transporters PlpA and GlnP

**DOI:** 10.1128/aac.00882-26

**Published:** 2026-10-05

**Authors:** Wenxin Zhang, Lu Zhang, Yanlin Sun, Mingzhong Zhao, Shujing Xu, Han Yu, Wenjing Wang, Jinmeng Liu, Wenyue Ma, Xiaoyu Wang, Ziyan Zhang, Yundong Sun

**Affiliations:** 1 Key Laboratory for Experimental Teratology of the Ministry of Education and Department of Microbiology, School of Basic Medical Sciences, Cheeloo College of Medicine, Shandong University 547479, Jinan, Shandong, China; 2 Department of Pathology, School of Basic Medical Sciences, Cheeloo College of Medicine, Shandong University 547479, Jinan, Shandong, China; Entasis, Big Bay, Michigan, USA

**Keywords:** *Helicobacter pylori*, biofilm, two-component system CrdRS, sub-inhibitory antibiotics, ABC transporter, reactive oxygen species

## Abstract

Biofilm formation by *Helicobacter pylori* is a major driver of antibiotic tolerance and treatment failure, yet the signaling pathways that trigger biofilm development under sub-inhibitory antibiotic pressure remain poorly understood. Here, we show that sub-MIC levels of metronidazole, amoxicillin, and ciprofloxacin potently induce dense *H. pylori* biofilms. Through transcriptomic and genetic analyses, we identify the two-component system CrdRS as a central signaling hub that orchestrates this response via the bidirectional regulation of two ATP-binding cassette (ABC) transporters. Phosphorylated CrdR binds to AC-rich promoter motifs to directly activate *plpA*, which encodes a substrate-binding protein that drives exopolysaccharide secretion and matrix assembly. Concomitantly, CrdR represses *glnP*, which encodes an inner-membrane permease, thereby relieving transcriptional inhibition of the L-asparaginase gene *ansB*. This derepression triggers aberrant reactive oxygen species (ROS) accumulation, which promotes oxidative stress-dependent biofilm maturation. Through phenotypic analysis of c*rdRS* deletion mutants, phosphorylation-defective point mutants (CrdR^D53A^ and CrdS^H173A^), and exogenous hydrogen peroxide (H₂O₂) induction, we demonstrate that CrdRS is required for mediating antibiotic-induced stress responses in a manner genetically separable from ROS sensing. Collectively, our findings establish a dual-mechanism model in which CrdRS orchestrates antibiotic-induced biofilm formation by simultaneously controlling matrix production and intracellular ROS generation. Notably, *glnP* expression was significantly lower in clinical multidrug-resistant isolates than in drug-sensitive ones, whereas *plpA* showed the opposite trend. These findings provide a mechanistic foundation for developing CrdRS-targeted strategies to combat biofilm-associated *H. pylori* infections.

## INTRODUCTION

*Helicobacter pylori*, a gastric pathogen that persistently colonizes approximately half of the global population, is etiologically linked to a spectrum of gastroduodenal diseases, including chronic gastritis, peptic ulcer disease, mucosa-associated lymphoid tissue lymphoma, and gastric adenocarcinoma (1). Current clinical management relies on combination antibiotic regimens, typically comprising a proton pump inhibitor plus two or three antimicrobial agents (2). However, the relentless rise of antibiotic resistance has steadily eroded eradication success rates (3). Global surveillance data indicate that primary resistance to clarithromycin, metronidazole, and levofloxacin has reached alarming levels in many regions; clarithromycin resistance alone exceeds 15% in most WHO regions, rendering standard triple therapy unacceptably ineffective (4, 5).

Beyond genetically encoded resistance, a growing body of clinical evidence implicates biofilm formation as a critical factor in treatment failure. In patients who have failed multiple eradication regimens, *H. pylori* is frequently found organized into dense biofilms on the gastric mucosa, and isolates from such patients exhibit significantly higher biofilm-forming capacity than those from treatment-naïve individuals (6, 7). Biofilms are surface-associated bacterial communities encased in a self-produced matrix of extracellular polymeric substances (EPS). This matrix acts as a physical diffusion barrier and creates metabolically dormant microenvironments, conferring phenotypic tolerance that can exceed planktonic susceptibility by 10- to 1,000-fold (8). Biofilm-mediated tolerance thus constitutes a major obstacle to successful eradication and a key driver of persistent infection (9, 10).

*H. pylori* biofilm development proceeds through sequential stages: initial attachment, microcolony formation, maturation, and dispersal (11). Planktonic cells first adhere to gastric epithelium or abiotic surfaces through flagellar motility and outer membrane adhesins, such as BabA, SabA, and HopQ (12–14); deletion of motility genes—*motB*, *fliM*, and *fliA*—severely impairs biofilm initiation (15, 16). Mature biofilms are structurally heterogeneous, comprising spiral and coccoid forms embedded in an EPS matrix rich in proteomannans, fucose, galactose, *N*-acetylglucosamine, eDNA, and proteins (8). The quorum-sensing molecule AI-2, synthesized by LuxS (17), modulates biofilm dynamics in a complex, concentration-dependent manner (18): paradoxically, luxS deletion mutants incapable of AI-2 production exhibit enhanced biofilm formation (18, 19), suggesting that AI-2 signaling helps regulate the planktonic-to-biofilm transition (18). Despite substantial progress in characterizing *H. pylori* biofilm structure and composition, the upstream signals and signaling pathways that trigger this transition remain largely undefined (8).

In the gastric niche, *H. pylori* faces continuous environmental stress—reactive oxygen and nitrogen species generated by host inflammatory responses, pH fluctuations, and nutrient limitation (11, 20, 21). Under such adverse conditions, adopting a biofilm lifestyle represents a critical adaptive strategy that enhances bacterial survival (8). During antibiotic therapy, pharmacokinetic constraints—including limited drug penetration through the gastric mucus layer, acid-catalyzed degradation, and uneven tissue distribution—often prevent sustained bactericidal concentrations at the infection site, creating exposure windows at sub-MICs (22, 23). Accumulating evidence indicates that sub-MIC antibiotics act not merely as ineffective antimicrobials, but as potent environmental signals that trigger bacterial stress responses and induce biofilm formation (24, 25). In *H. pylori*, Krzyżek et al. recently showed that continuous exposure to sub-MICs of metronidazole or levofloxacin accelerates autoaggregation, enhances EPS production, and increases biofilm dimensions, whereas clarithromycin exerts the opposite effect (26). This phenomenon mirrors observations in other clinically significant pathogens—*Staphylococcus aureus*, *Pseudomonas aeruginosa*, and *Escherichia coli*—where sub-lethal antibiotic concentrations promote biofilm development, suggesting a phylogenetically conserved adaptive strategy (27, 28). Nevertheless, the molecular machinery—specifically, the sensory systems and downstream regulatory cascades—that enables *H. pylori* to perceive sub-MIC antibiotics as signals and translate them into a biofilm developmental program has yet to be systematically elucidated.

Bacteria rely on two-component systems (TCS) as primary signal-transduction mechanisms for sensing and responding to environmental changes. A canonical TCS consists of a membrane-anchored sensor histidine kinase (HK) and a cognate cytoplasmic response regulator (RR). Upon stimulus perception, the HK undergoes ATP-dependent autophosphorylation at a conserved histidine residue and subsequently transfers the phosphoryl group to an aspartate residue on the RR. Phosphorylation induces a conformational change that activates the RR’s DNA-binding domain, enabling sequence-specific promoter recognition and target gene modulation (29). The *H. pylori* genome encodes a limited TCS repertoire that governs critical adaptive responses: ArsRS senses acidic pH and regulates acid-adaptive genes, including the urease cluster (30); FlgRS controls flagellar biosynthesis and motility (31); and CrdRS (HP1364/HP1365) was originally characterized as a copper-sensing system that maintains metal ion homeostasis by activating the copper resistance gene *crdA* (32) and was later shown to also respond to nitrosative stress (33). Notably, several TCSs have been implicated in biofilm regulation. ArsRS negatively regulates biofilm development: *arsS* deletion upregulates the outer membrane protein HomB and enhances biofilm formation (34, 35). FlgR, via inhibition of the alternative sigma factor σ⁵⁴, represses the molybdate transport system ModABD, thereby suppressing biofilm formation; reactive oxygen species (ROS)-induced allosteric inhibition of the nickel-responsive regulator NikR relieves this FlgR-mediated repression, providing a mechanistic link between oxidative stress and biofilm induction (36). These findings establish that TCSs can integrate diverse environmental cues—pH, metal ions, oxidative stress—to control the biofilm developmental switch. However, whether any TCS senses antibiotic signals to mediate sub-MIC-induced biofilm formation has remained an open question.

In this study, we systematically investigated the signaling network underlying sub-MIC antibiotic-induced biofilm formation in *H. pylori*. We demonstrate that CrdRS functions as a central antibiotic-sensing hub that orchestrates biofilm development through a dual regulatory mechanism: phosphorylated CrdR directly activates *plpA* to drive exopolysaccharide secretion and matrix assembly, while concurrently repressing *glnP*, thereby triggering *ansB*-dependent ROS accumulation and oxidative stress-driven biofilm maturation.

## RESULTS

### Sub-inhibitory concentrations of antibiotics induce biofilm formation in *H. pylori in vitro*

**Fig 1:**
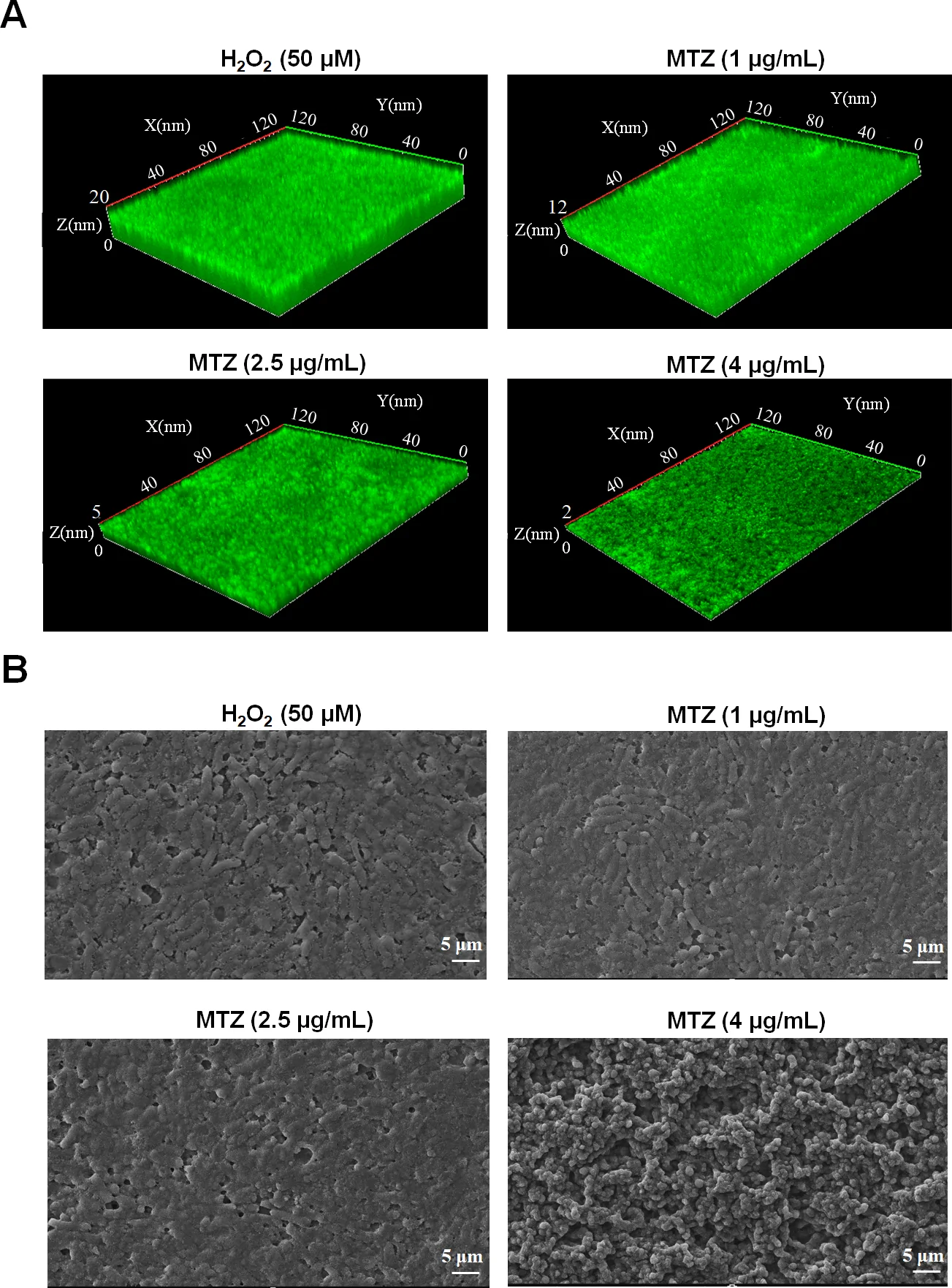
Sub-inhibitory concentrations of MTZ induce *H. pylori* biofilm formation. WT *H. pylori* strain 26695 was cultured on nitrocellulose membranes for 72 h in the presence of MTZ at the indicated concentrations (0, 1, 2.5, and 4 μg/mL). Untreated WT (0 μg/mL) served as the negative control, and WT exposed to 50 μM H₂O₂ served as the positive control. (**A**) Representative CLSM images of biofilms stained with SYTO 9 (green) to visualize viable bacteria. (**B**) Representative SEM images showing biofilm ultrastructure. Scale bar, 5 μm. Data are representative of three independent experiments.

To determine whether low antibiotic concentrations induce biofilm formation in *H. pylori*, we selected three clinically relevant antibiotics—metronidazole (MTZ), amoxicillin (AMX), and ciprofloxacin (CIP). For each, we established three concentration gradients—two sub-MIC levels and one at the MIC—and examined their effects on biofilm formation by the wild-type strain 26695 (WT) using confocal laser scanning microscopy (CLSM) and scanning electron microscopy (SEM). Sub-inhibitory MTZ at 1 μg/mL induced the most robust biofilm formation, comparable to that elicited by low-concentration H₂O₂ (positive control). CLSM revealed that biofilms formed under 1 μg/mL MTZ exhibited a highly compact architecture with markedly reduced microchannels and interbacterial spaces, and were substantially thicker than those formed under other conditions (Fig. 1A). SEM confirmed that the extracellular matrix of these biofilms was densely organized (Fig. 1B). As MTZ concentration increased, biofilm biomass decreased significantly (Fig. S1A); at the MIC, biofilm thickness was markedly reduced (Fig. 1A) and matrix content substantially diminished (Fig. 1B). Similarly, sub-MICs of AMX (1/32 μg/mL) and CIP (1/16 μg/mL) both induced dense, intact biofilms (Fig. S1B, C, S2 and S3).

### PlpA is essential for sub-inhibitory antibiotic-induced biofilm formation

To elucidate the molecular mechanisms underlying sub-inhibitory antibiotic-induced biofilm formation, we performed transcriptomic analysis on WT biofilm cells treated with the three antibiotics. KEGG pathway enrichment revealed that differentially expressed genes were significantly enriched in “ATP-binding cassette (ABC) transporters” and “alanine, aspartate, and glutamate metabolism” (Fig. S4A). Notably, heatmap and quantitative real-time PCR (qRT-PCR) analyses confirmed that, compared with planktonic WT cells cultured without antibiotics, the outer-membrane substrate-binding protein gene *plpA* (*hp1564*) was markedly upregulated, while the inner-membrane amino acid permease gene cluster *glnP* (*hp1169–hp1170*) was significantly downregulated (Fig. S4B and C).

**Fig 2:**
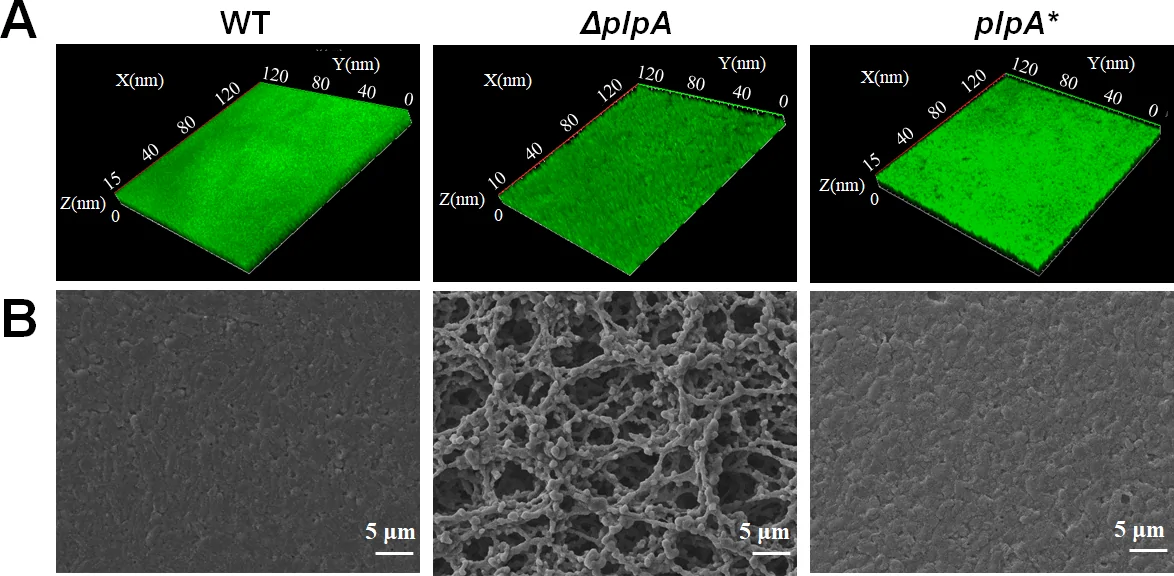
PlpA is essential for sub-inhibitory MTZ-induced biofilm formation in *H. pylori*. WT, *ΔplpA*, and plpA* complemented strains were cultured on nitrocellulose membranes containing MTZ (1 μg/mL) for 3 days to allow mature biofilm development. (**A**) Representative CLSM images of biofilms stained with SYTO 9 (green). (**B**) Representative SEM images of biofilm architecture. Scale bar, 5 μm. Data are representative of three independent experiments.

To verify whether PlpA mediates sub-inhibitory antibiotic-induced biofilm formation, we constructed a *plpA* deletion mutant (*ΔplpA*) and its complementation strain (plpA*). Growth curve analysis revealed no significant growth defect in the *ΔplpA* strain (Fig. S5A). After 72 h of induction with sub-inhibitory MTZ, we assessed biofilm formation by CLSM and SEM. The WT and plpA* strains formed structurally compact, intact biofilms with abundant extracellular matrix, whereas the *ΔplpA* strain displayed virtually no intact biofilm architecture, reduced extracellular matrix, and increased inter-membrane void spaces (Fig. 2). Quantitative biofilm assays further confirmed significantly reduced biomass in the *ΔplpA* mutant (Fig. S1D). Consistent results were also obtained with AMX and CIP (Fig. S1E, F, S6 and S7). Moreover, the functional requirement for PlpA in biofilm formation was further confirmed in a clinical context, as deletion of *plpA* in the H57 clinical isolate (H57*ΔplpA*) also resulted in markedly reduced biofilm formation compared with the parental H57 strain (Fig. S8).

### Downregulation of GlnP mediates biofilm formation under sub-inhibitory antibiotic stress

**Fig 3:**
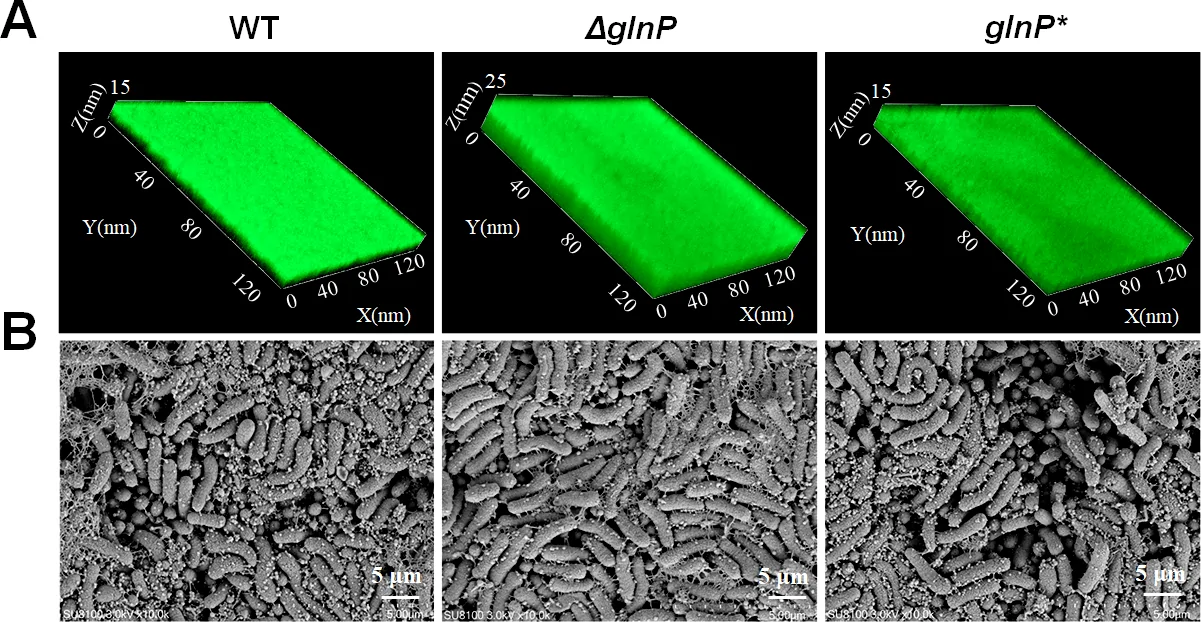
Deletion of *glnP* enhances sub-inhibitory MTZ-induced biofilm formation in *H. pylori*. WT, *ΔglnP*, and glnP* complemented strains were cultured on nitrocellulose membranes containing MTZ (1 μg/mL) for 3 days. (**A**) Representative CLSM images of biofilms stained with SYTO 9 (green). (**B**) Representative SEM images showing biofilm ultrastructure. Scale bar, 5 μm. Data are representative of three independent experiments.

To investigate whether GlnP participates in biofilm regulation, we generated a *glnP* deletion mutant (*ΔglnP*) and its complementation strain (glnP*). Growth curve analysis showed that the mutant and complementation strains grew comparably to WT (Fig. S5B). Following 24 h of treatment with sub-inhibitory MTZ, CLSM and SEM analyses revealed that the *ΔglnP* mutant formed substantially denser and thicker biofilms with increased extracellular matrix content compared with WT and glnP* (Fig. 3A and B). Quantitative biofilm assays further confirmed significantly increased biomass in the *ΔglnP* mutant (Fig. S1G). Similar results were obtained with AMX and CIP (Fig. S9 and S10). In the clinical isolate H57, deletion of *glnP* (H57*ΔglnP*) also resulted in markedly enhanced biofilm formation compared with the parental H57 strain (Fig. S11).

### The CrdRS two-component system is required for sub-inhibitory antibiotic-induced biofilm formation

To explore how *plpA* expression is elevated and *glnP* expression suppressed during biofilm formation, we performed transcriptomic analysis to identify differentially expressed regulatory genes in biofilm cells. Compared with planktonic wild-type cells cultured without antibiotic treatment, hierarchical clustering and heatmap visualization revealed marked upregulation of the transcriptional regulator gene *hp1365* (Fig. S12) in biofilm cells treated with sub-inhibitory antibiotics. *hp1365* encodes CrdR, the response regulator of the CrdRS two-component system (32). Given this high expression and the established signaling roles of TCSs, we hypothesized that CrdRS participates in antibiotic-induced biofilm formation.

**Fig 4:**
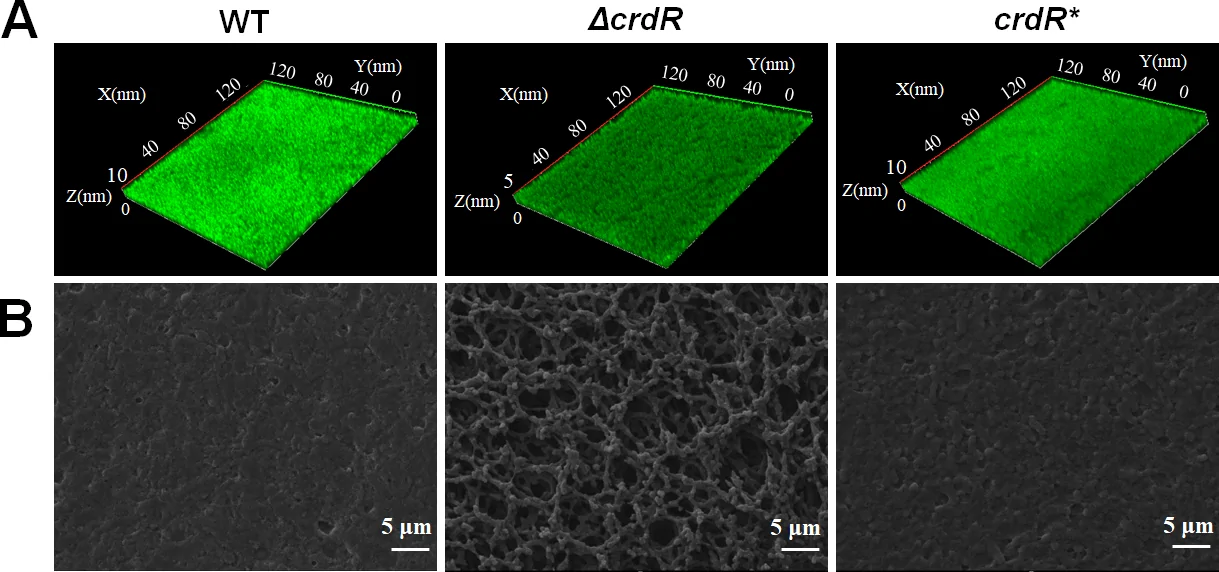
CrdR is required for sub-inhibitory MTZ-induced biofilm formation in *H. pylori*. WT, *ΔcrdR*, and crdR* complemented strains were cultured on nitrocellulose membranes containing MTZ (1 μg/mL) for 3 days. (**A**) Representative CLSM images of biofilms stained with SYTO 9 (green). (**B**) Representative SEM images of biofilm architecture. Scale bar, 5 μm. Data are representative of three independent experiments.

**Fig 5:**
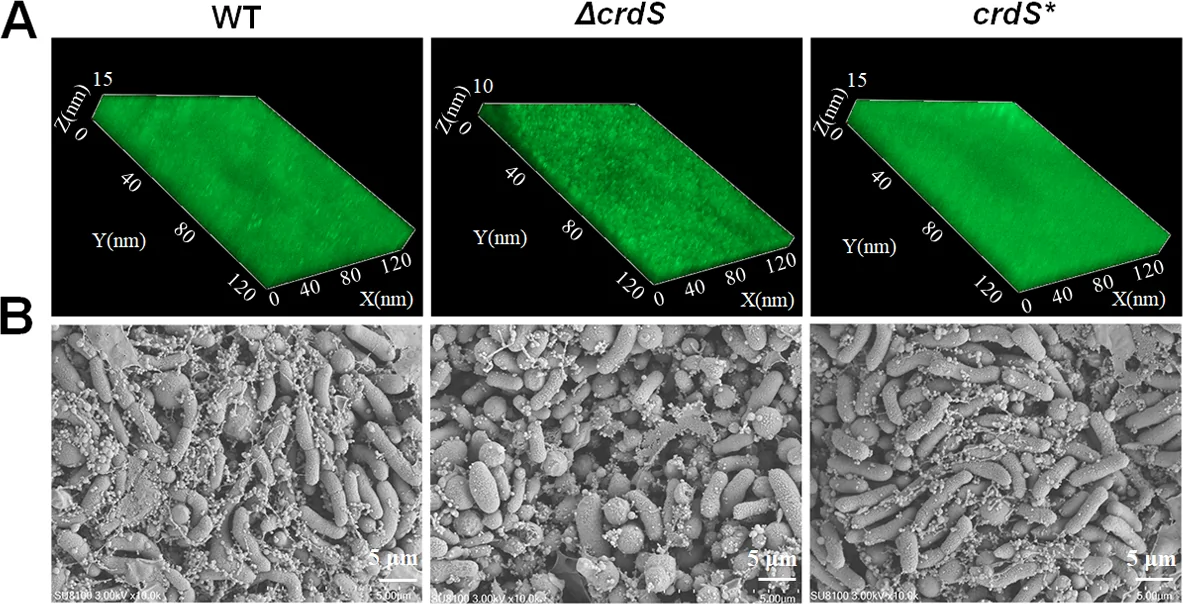
CrdS is required for sub-inhibitory MTZ-induced biofilm formation in *H. pylori*. WT, *ΔcrdS*, and crdS* complemented strains were cultured as described in Fig. 4. (**A**) Representative CLSM images of biofilms stained with SYTO 9 (green). (**B**) Representative SEM images of biofilm ultrastructure. Scale bar, 5 μm. Data are representative of three independent experiments.

To test this, we constructed *crdRS* deletion mutants (*ΔcrdR* and *ΔcrdS*), complementation strains (crdR* and crdS*), and phosphorylation-defective point mutants (CrdR^D53A^ and CrdS^H173A^). All strains exhibited growth kinetics comparable to WT (Fig. S5C through E). Following induction with sub-inhibitory antibiotics (MTZ, AMX, or CIP), we examined biofilm morphology by CLSM and SEM. Compared with WT, the *ΔcrdR*, *ΔcrdS*, CrdR^D53A^, and CrdS^H173A^ strains all displayed reduced biofilm thickness and biomass, increased intercellular microchannels and void spaces, rougher surface textures, and diminished extracellular matrix (Fig. 4 and 5; Fig. S1H through K and S13 to S16). In contrast, the complementation strains (crdR* and crdS*) formed biofilms comparable to those of WT (Fig. 4 and 5).

### CrdR upregulates the ABC transporter PlpA to promote antibiotic-induced biofilm formation

**Fig 6:**
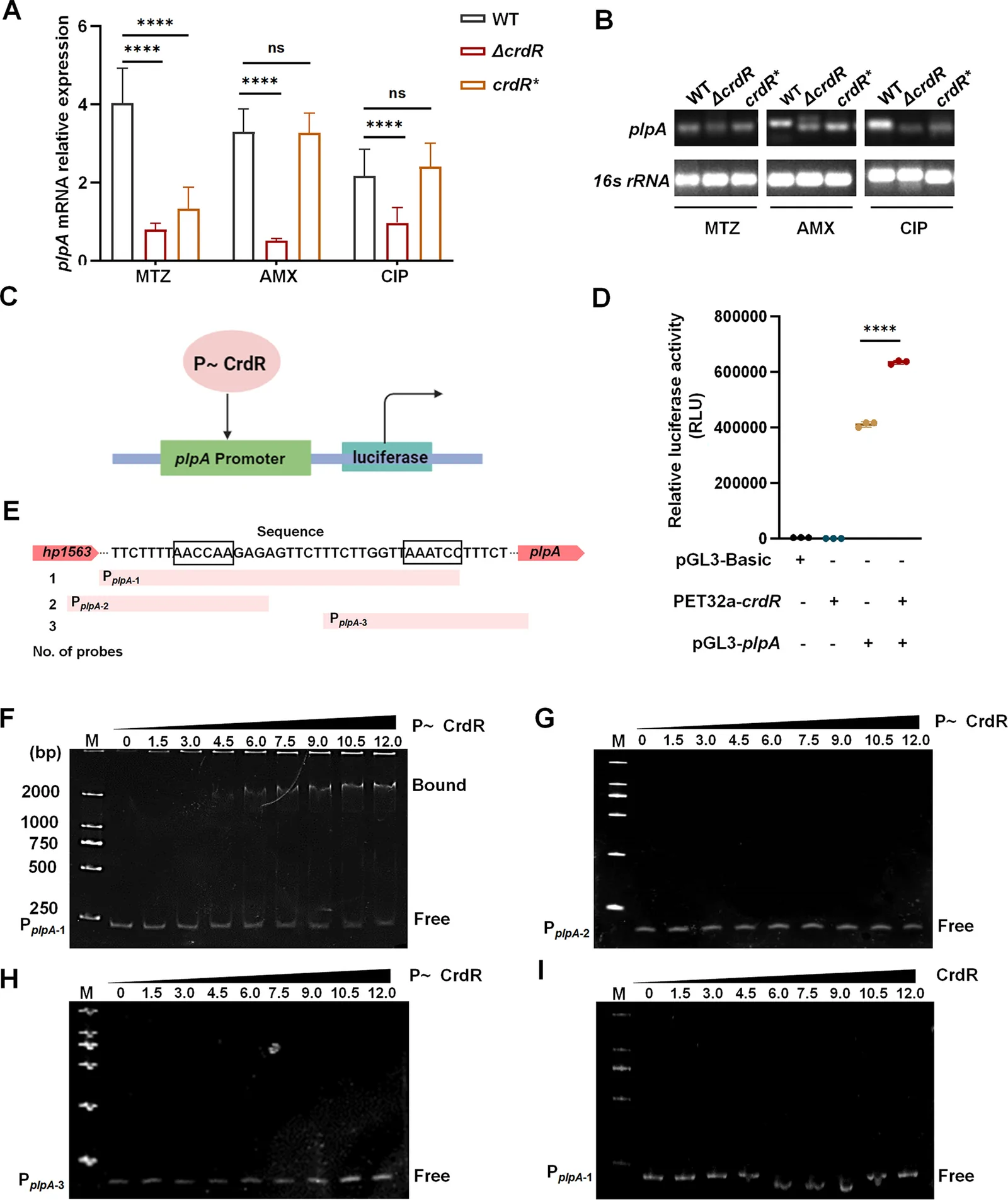
Phosphorylated CrdR directly binds the *plpA* promoter to activate its transcription. (**A**) Relative *plpA* mRNA levels in WT, *ΔcrdR*, and crdR* strains following treatment with sub-inhibitory concentrations of MTZ (1 μg/mL), AMX (1/32 μg/mL), or CIP (1/16 μg/mL), determined by qRT-PCR. (**B**) Semi-quantitative RT-PCR analysis of *plpA* expression in the indicated strains. (**C**) Schematic of the luciferase reporter assay. (**D**) Luciferase activity in *E. coli* BL21 co-transformed with the reporter plasmid pGL3-OP*_plpA_* and the CrdR expression plasmid pET32a-*crdR* in the combinations indicated. Empty pGL3-Basic and pET32a vectors served as controls. (**E**) Schematic representation of electrophoretic mobility shift assay (EMSA) probes spanning the *plpA* promoter region. The intergenic region between *hp1563* and *plpA* contains two AC-rich motifs (shaded boxes). Probe P*_plpA_*_-1_ (~100 bp) encompasses both motifs; probes P*_plpA_*_-2_ and P*_plpA_*_-3_ (~70 bp each) contain only the upstream or downstream motif, respectively. (**F–I**) EMSA analysis of CrdR binding. (**F**) Phosphorylated CrdR (P~CrdR) binds to probe P*_plpA_*_-1_ containing dual AC-rich motifs, producing a shifted complex (bound). (**G and H**) Phosphorylated CrdR does not bind to probes P*_plpA_*_-2_ or P*_plpA_*_-3_ containing single AC-rich motifs. (**I**) Unphosphorylated CrdR does not bind to probe P*_plpA_*_-1_. Free probe, unbound DNA. CrdR protein concentrations in lanes 1 to 9: 0, 1.5, 3, 4.5, 6, 7.5, 9, 10.5, and 12 μM. Data in panels **A** and **D** are presented as mean ± SD; statistical significance: ****, *P* < 0.0001; ns, not significant. All experiments were performed with three independent biological replicates.

To verify whether CrdR regulates PlpA, we used qRT-PCR and semi-quantitative RT-PCR to measure *plpA* mRNA levels in biofilm cells of the *ΔcrdR* and crdR* strains. Under sub-inhibitory antibiotic treatment, *plpA* expression was markedly reduced in the *ΔcrdR* strain relative to WT (Fig. 6A and B), indicating that CrdR positively regulates *plpA* transcription. As *E. coli* can phosphorylate heterologously expressed response regulators (37), we performed luciferase reporter assays in this host to assess the regulatory roles of CrdR on the *plpA* promoter. *E. coli* co-transformed with pGL3-OP*_plpA_* and pET32a-*crdR* exhibited significantly higher luciferase activity than cells harboring pGL3-OP*_plpA_* alone (Fig. 6C and D), showing that CrdR activates *plpA* expression.

We next investigated whether phosphorylated CrdR directly binds the *plpA* promoter using an electrophoretic mobility shift assay (EMSA). We designed three DNA probes: probe P*_plpA_*_-1_ containing two AC-rich regions (~100 bp); probe P*_plpA_*_-2_ containing the upstream AC-rich region (~70 bp); and probe P*_plpA_*_-3_ containing the downstream AC-rich region (~70 bp) (Fig. 6E). Phosphorylated CrdR bound to probe P*_plpA_*_-1_, which harbors dual AC-rich motifs (Fig. 6F), but did not bind to probes P*_plpA_*_-2_ or P*_plpA_*_-3_, which contain a single AC-rich region (Fig. 6G and H). Consistent with the established paradigm for OmpR-family response regulators (38), and given that CrdR belongs to this family (32), unphosphorylated CrdR failed to bind any probe (Fig. 6I), and the phosphorylation-site mutant CrdR^D53A^ was unable to form complexes with the *plpA* dual-site probes (Fig. S17A). These results demonstrate that phosphorylated CrdR specifically binds the AC-rich region of the *plpA* promoter to activate transcription.

### CrdR downregulates the ABC transporter GlnP to promote antibiotic-induced biofilm formation

**Fig 7:**
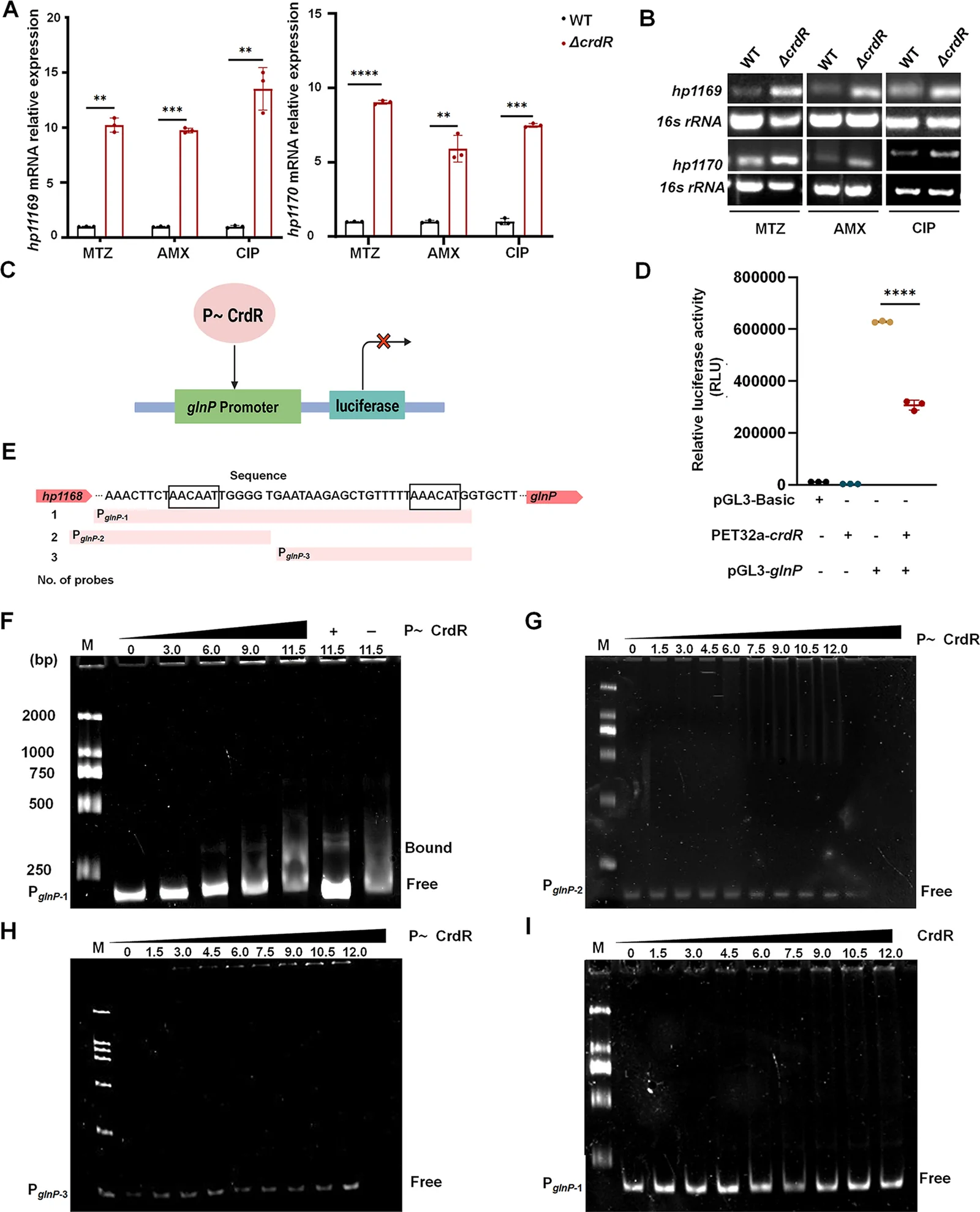
Phosphorylated CrdR directly binds the *glnP* promoter to repress its transcription. (**A**) Relative expression of the *glnP* gene cluster (*hp1169–hp1170*) in WT and *ΔcrdR* strains treated with sub-inhibitory MTZ, AMX, or CIP, determined by qRT-PCR. (**B**) Semi-quantitative RT-PCR analysis of *glnP* expression in the *ΔcrdR* strain. (**C**) Schematic of the luciferase reporter assay. (**D**) Luciferase activity in *E. coli* BL21 co-transformed with pGL3-OP*_glnP_* and pET32a-*crdR* in the indicated combinations. (**E**) Schematic representation of EMSA probes spanning the *glnP* promoter. Blue boxes indicate putative CrdR-binding sites. Probe P*_glnP_*_-1_ contains dual binding sites; probes P*_glnP_*_-2_ and P*_glnP_*_-3_ contain only the upstream or downstream single site, respectively. (**F–I**) EMSA analysis of CrdR binding to the *glnP* promoter. (**F**) Phosphorylated CrdR (P~CrdR) specifically binds probe P*_glnP_*_-1_ containing dual sites. Lane 6, *crdA* promoter probe (positive control); lane 7, random sequence probe (negative control). CrdR protein concentrations in lanes 1 to 7: 0, 3, 6, 9, 11.5, 11.5, and 11.5 μM. (**G and H**) P~CrdR does not bind to probes P*_glnP_*_-2_ or P*_glnP_*_-3_ containing single sites. (**I**) Unphosphorylated CrdR does not bind probe P*_glnP_*_-1_. Bound, DNA-protein complex; free probe, unbound DNA. (**G–I**) CrdR protein concentrations in lanes 1 to 9: 0, 1.5, 3, 4.5, 6, 7.5, 9, 10.5, and 12 μM. Data in panels **A** and **B** are presented as mean ± SD; statistical significance: **, *P* < 0.01; ***, *P* < 0.001; ****, *P* < 0.0001; ns, not significant. All experiments were performed with three independent biological replicates.

To assess whether CrdR regulates GlnP, we used qRT-PCR and RT-PCR to measure *glnP* (*hp1169–hp1170*) mRNA expression in *ΔcrdR* biofilm cells. *glnP* expression was markedly upregulated in the *ΔcrdR* strain compared with WT (Fig. 7A and B), indicating that CrdR negatively regulates *glnP*. Luciferase reporter analysis further showed that *E. coli* co-transformed with pGL3-OP*_glnP_* and pET32a-*crdR* exhibited significantly reduced luciferase activity relative to cells expressing pGL3-OP*_glnP_* alone (Fig. 7C and D), confirming that CrdR represses *glnP* expression.

We next performed EMSA to test direct binding. We designed three probes: P*_glnP_*_-1_ containing two AC-rich regions (~180 bp); P*_glnP_*_-2_ containing the upstream AC-rich region (~120 bp); and P*_glnP_*_-3_ containing the downstream AC-rich region (~70 bp) (Fig. 7E). Phosphorylated CrdR specifically bound probe P*_glnP_*_-1_ containing dual AC-rich motifs (Fig. 7F), but showed no significant interaction with the single-site probes (Fig. 7G and H). Unphosphorylated CrdR failed to bind the *glnP* promoter probes (Fig. 7I), and the phosphorylation-site mutant CrdR^D53A^ was unable to form complexes with the *glnP* dual-site probes (Fig. S17B). These results confirm that phosphorylation at Asp53 is essential for CrdR DNA-binding activity.

### Deletion of *plpA* reduces polysaccharide content in the biofilm extracellular matrix

**Fig 8:**
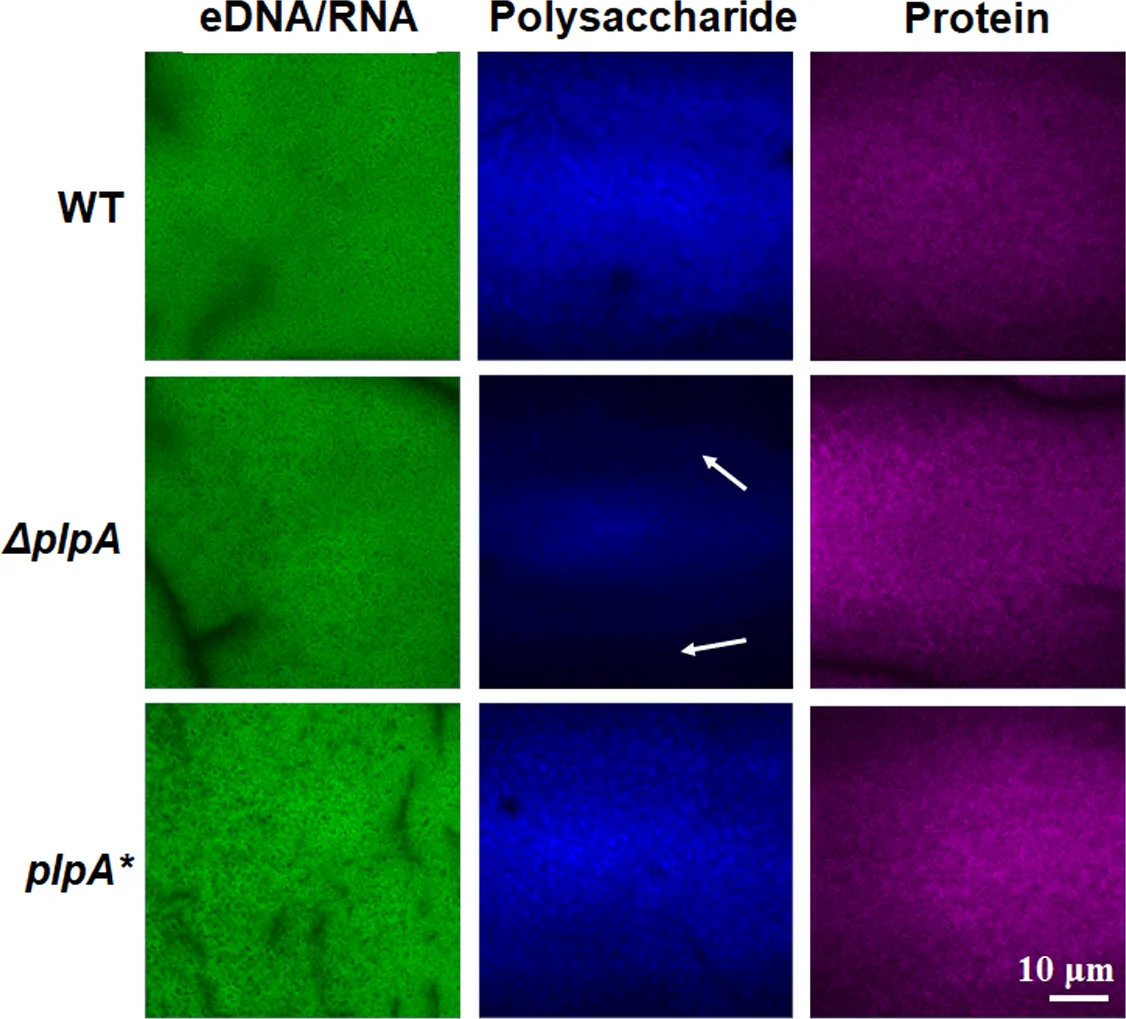
Deletion of *plpA* reduces polysaccharide content in the biofilm extracellular matrix. WT, *ΔplpA*, and plpA* biofilms were cultured on nitrocellulose membranes for 3 days and stained for EPS components. eDNA/RNA was visualized with propidium iodide (PI), exopolysaccharides with Calcofluor White, and extracellular proteins with SYPRO Ruby. Representative CLSM images are shown. White arrows indicate regions with reduced fluorescence signal, suggesting decreased polysaccharide content in the EPS of the *ΔplpA* mutant compared with WT and plpA*. Scale bar, 10 μm. Data are representative of three independent experiments.

To determine why *plpA* deletion impairs extracellular matrix formation, we compared the major EPS components—proteins, eDNA/RNA, and polysaccharides—in WT, *ΔplpA*, and plpA* biofilms by specific staining. Polysaccharide content was markedly reduced in the *ΔplpA* biofilm EPS compared with WT, whereas eDNA/RNA content was only slightly decreased. The EPS composition of the plpA* strain was comparable to that of WT (Fig. 8).

### *glnP* deletion upregulates *ansB*, driving ROS-dependent biofilm formation

To elucidate the metabolic mechanisms through which *glnP* deletion promotes biofilm formation, we performed transcriptomic (RNA-seq) analysis on WT and *ΔglnP* strains under sub-inhibitory antibiotic stress. KEGG enrichment analysis showed that upregulated genes in the *ΔglnP* strain were significantly enriched in TCA cycle and amino acid metabolism pathways (Fig. S18A). Notably, the L-asparaginase gene *ansB* (*hp0723*), which links these two metabolic pathways, was markedly upregulated (Fig. S18B). Aspartate generated by AnsB-catalyzed deamination can enter the TCA cycle via transamination, consuming α-ketoglutarate (α-KG) (39, 40). Depletion of the α-KG pool may impair the cell’s metabolic flux and redox balance, as α-KG itself possesses direct ROS-scavenging activity (40, 41). This, in turn, could lead to an accumulation of intracellular ROS. Given that low-level ROS are known to induce biofilm formation in *H. pylori* (20), we hypothesized that *glnP* deletion activates *ansB*, causing oxidative stress and ROS-dependent biofilm assembly.

**Fig 9:**
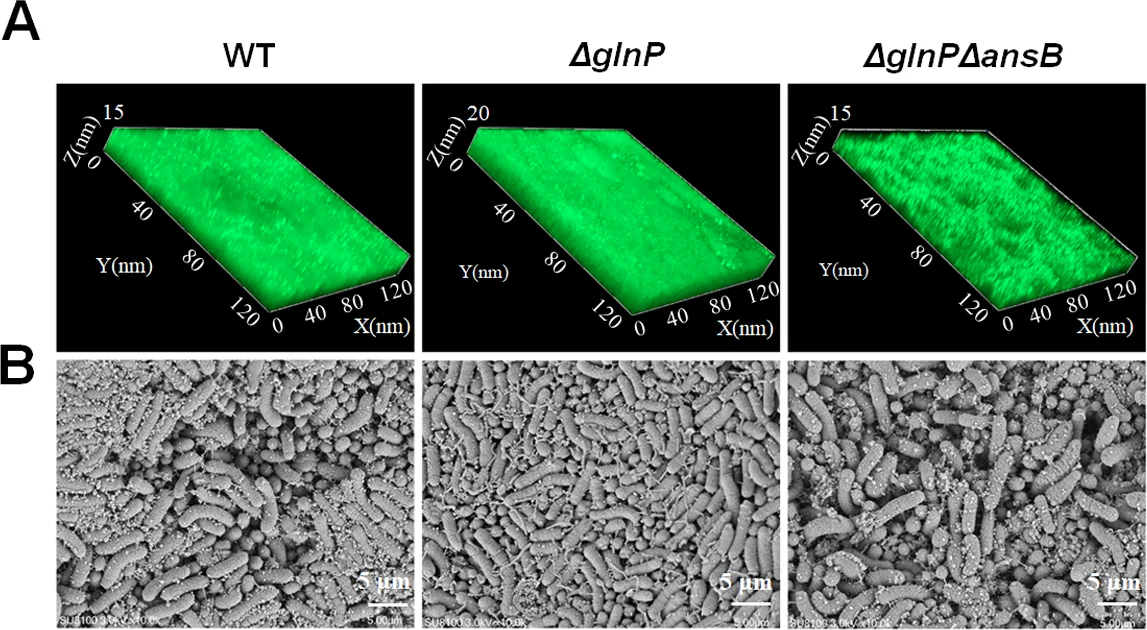
Deletion of *ansB* reverses the excessive biofilm formation phenotype of the *ΔglnP* mutant under sub-inhibitory MTZ induction. WT, *ΔglnP*, and *ΔglnPΔansB* strains were cultured on nitrocellulose membranes containing MTZ (1 μg/mL) for 3 days. (**A**) Three-dimensional CLSM reconstructions showing differences in biofilm thickness (Z-axis) among the indicated strains. (**B**) Representative SEM images of biofilm microarchitecture. Scale bar, 5 μm. Data are representative of three independent experiments.

To verify the role of *ansB*, we constructed a *ΔglnPΔansB* double mutant. Growth curve analysis showed that the double mutant grew comparably to WT and the *ΔglnP* single mutant (Fig. S5F). We compared biofilm formation induced by sub-inhibitory concentrations of MTZ, AMX, and CIP among WT, *ΔglnP*, and *ΔglnPΔansB* strains. The *ΔglnPΔansB* strain exhibited significantly reduced biofilm thickness, loosely arranged bacterial architecture, diminished EPS compared with the *ΔglnP* strain (Fig. 9; Fig. S19 and S20), and decreased biofilm biomass (Fig. S1I), indicating that deletion of *ansB* effectively reversed the excessive biofilm phenotype conferred by *glnP* deletion.

### Upregulation of *ansB* induces intracellular ROS accumulation, promoting biofilm formation

**Fig 10:**
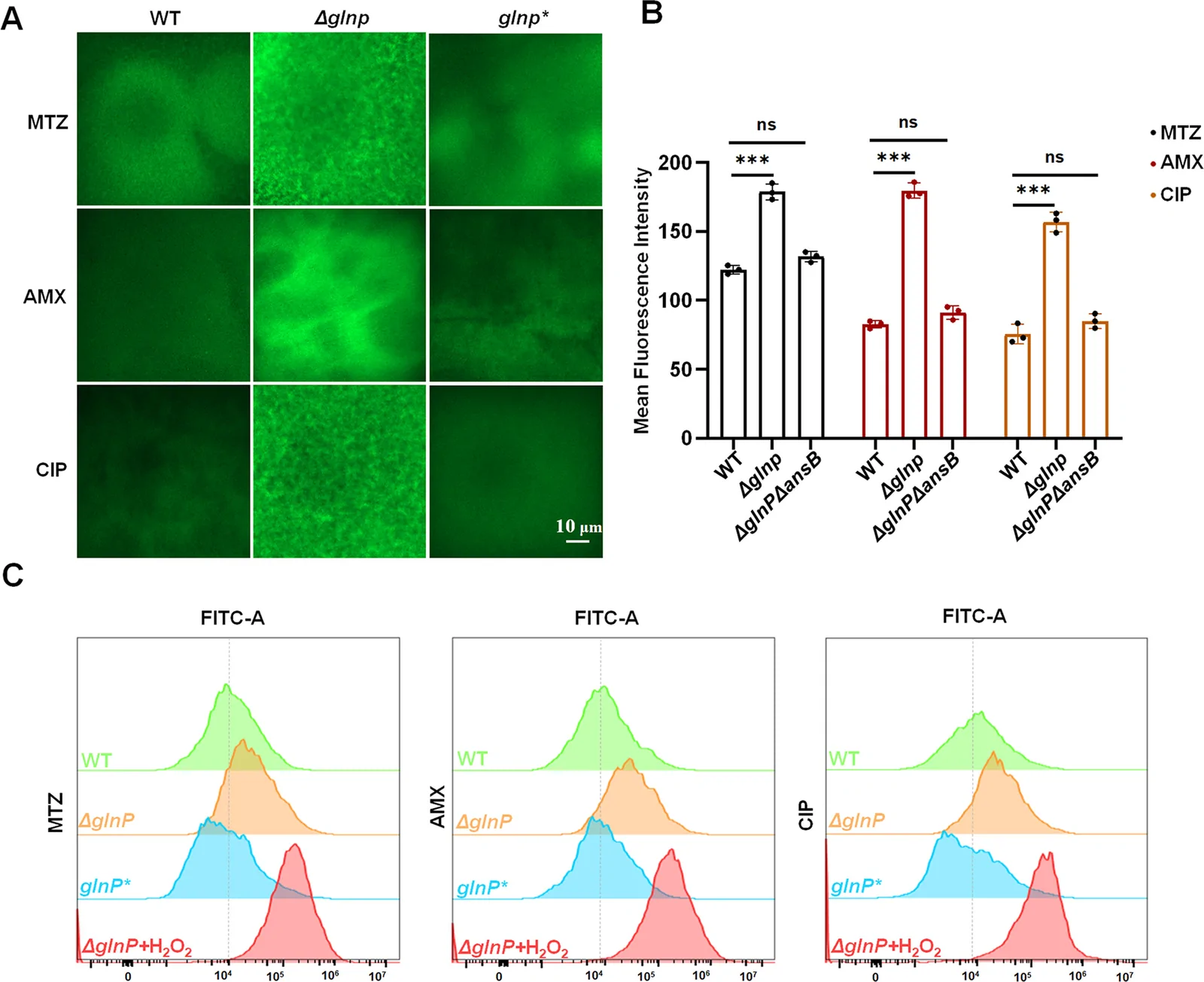
Deletion of *glnP* leads to intracellular ROS accumulation in *H. pylori* biofilms under sub-inhibitory antibiotic treatment. WT, *ΔglnP*, and glnP* strains were cultured with sub-inhibitory concentrations of MTZ (1 μg/mL), AMX (1/32 μg/mL), or CIP (1/16 μg/mL) for 3 days and stained with the ROS-sensitive fluorescent probe DCFH-DA. (**A**) Representative CLSM images showing intracellular ROS levels (green fluorescence). Scale bar, 10 μm. (**B**) Quantification of relative fluorescence intensity from CLSM images. (**C**) Flow cytometric analysis of intracellular ROS levels. Histogram peaks: green, WT; orange, *ΔglnP*; blue, glnP*; red, H₂O₂ (50 μM) positive control. Data in panels **B** and **C** are presented as mean ± SD; statistical significance: ***, *P* < 0.001; ns, not significant. All experiments were performed with three independent biological replicates.

**Fig 11:**
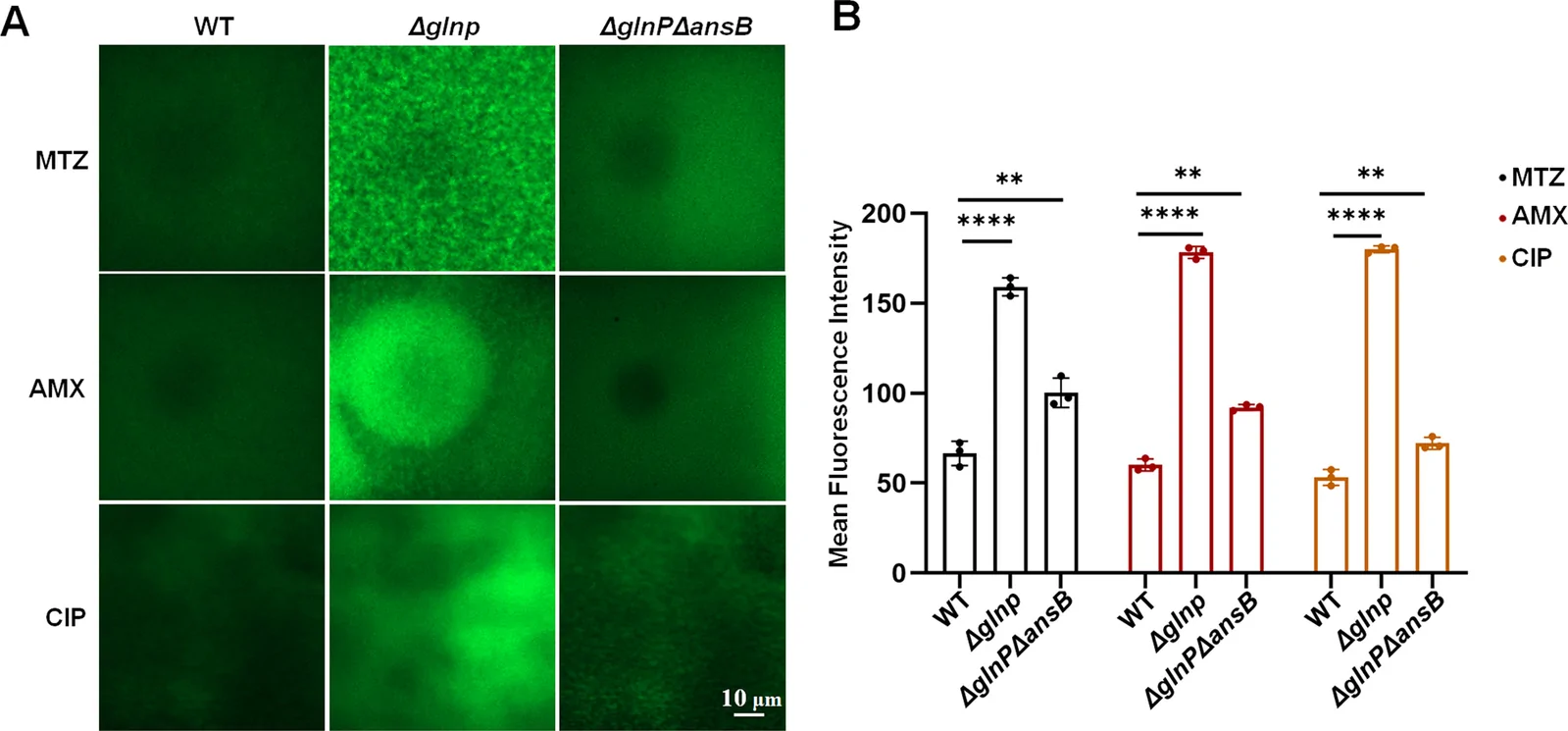
Deletion of *ansB* attenuates intracellular ROS accumulation in the *ΔglnP* mutant under sub-inhibitory antibiotic treatment. WT, *ΔglnP*, and *ΔglnPΔansB* strains were cultured with sub-inhibitory MTZ, AMX, or CIP for 3 days and stained with DCFH-DA. (**A**) Representative CLSM images of intracellular ROS (green fluorescence). Scale bar, 10 μm. (**B**) Quantification of relative fluorescence intensity from CLSM images. Data are presented as mean ± SD; statistical significance: **, *P* < 0.01; ****, *P* < 0.0001; ns, not significant. All experiments were performed with three independent biological replicates.

To test whether *ansB* upregulation leads to increased intracellular ROS, we used the DCFH-DA fluorescent probe to monitor endogenous ROS levels in biofilm cells following sub-inhibitory antibiotic treatment. Confocal microscopy revealed intense green fluorescence in *ΔglnP* biofilm cells, whereas WT and complementation (glnP*) strains displayed weak and comparable signals (Fig. 10A and B). Flow cytometric quantification confirmed a rightward shift of the fluorescence peak in the *ΔglnP* strain, indicating markedly elevated ROS levels, while the glnP* complementation strain reverted to WT baseline (Fig. 10C). In contrast, the *ΔglnPΔansB* double mutant exhibited dramatically attenuated fluorescence, returning essentially to WT levels (Fig. 11). This phenotypic rescue demonstrates that the ROS accumulation triggered by *glnP* deletion is critically dependent on *ansB* upregulation.

To further validate that ROS accumulation drives biofilm formation, we supplemented catalase during biofilm induction. Catalase treatment significantly inhibited biofilm formation in *ΔglnP*, *ΔglnPΔansB*, and WT strains in a concentration-dependent manner (Fig. S21).

### CrdRS regulates biofilm formation by specifically responding to antibiotic signals rather than ROS

**Fig 12:**
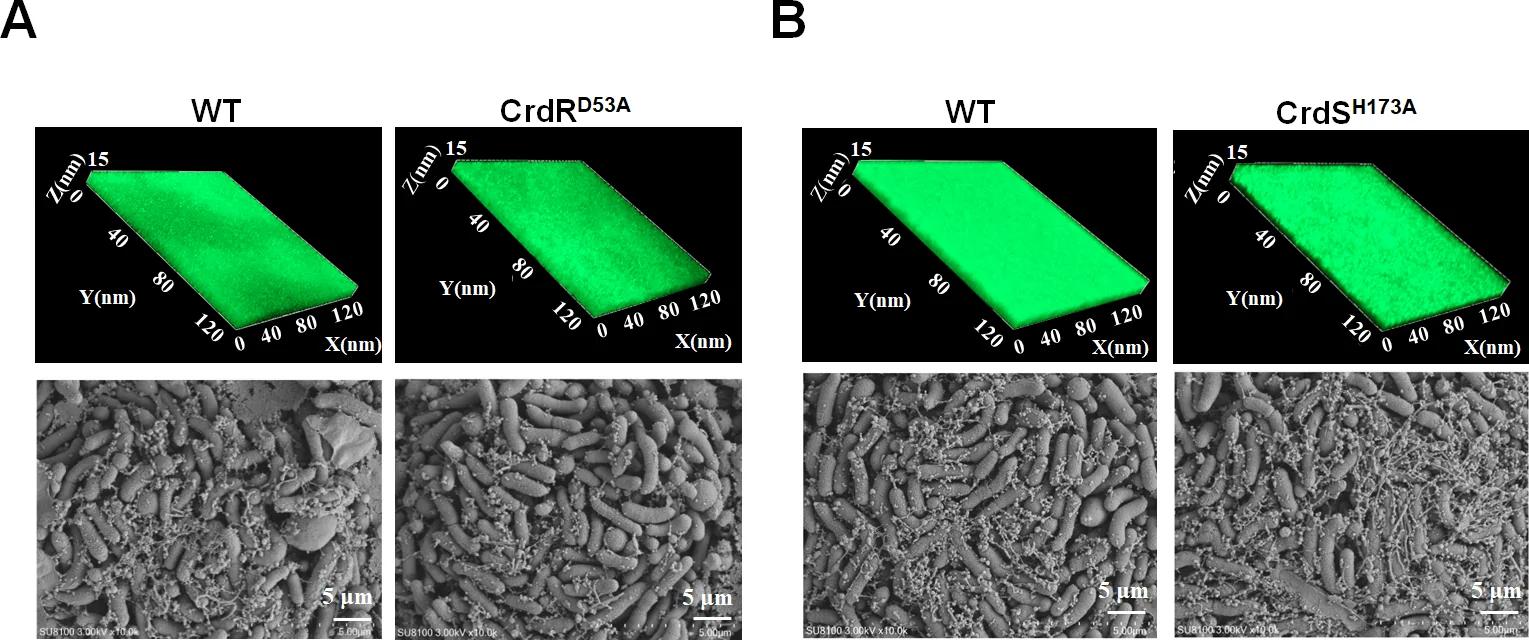
Phosphorylation-defective CrdRS mutants retain the capacity for H₂O₂-induced biofilm formation. (**A and B**) Biofilm morphology of WT versus CrdR^D53A^ (**A**) or CrdS^H173A^ (**B**) mutant strains following induction with 50 μM H₂O₂. Top panels: three-dimensional CLSM reconstructions showing biofilm thickness (Z-axis). Bottom panels: SEM images showing biofilm microstructure. Data are representative of three independent experiments.

Given that two-component systems can also sense endogenous signals, we asked whether CrdRS recognizes antibiotic molecules or is activated indirectly by ROS generated under antibiotic stress. We compared biofilm formation under H₂O₂ induction between WT and the phosphorylation-defective mutants CrdR^D53A^ and CrdS^H173A^ using CLSM and SEM. All strains—including the mutants—formed normal biofilms under direct H₂O₂ exposure (Fig. 12). This demonstrates that phosphorylation-site mutations in CrdRS do not impair H₂O₂-induced biofilm formation, indicating that CrdRS activation under antibiotic stress is genetically separable from ROS sensing. Thus, CrdRS is required for transduction of antibiotic-induced signals rather than functioning as a downstream ROS sensor.

### Differential expression of *glnP* and *plpA* in clinical multidrug-resistant and drug-sensitive isolates

**Fig 13:**
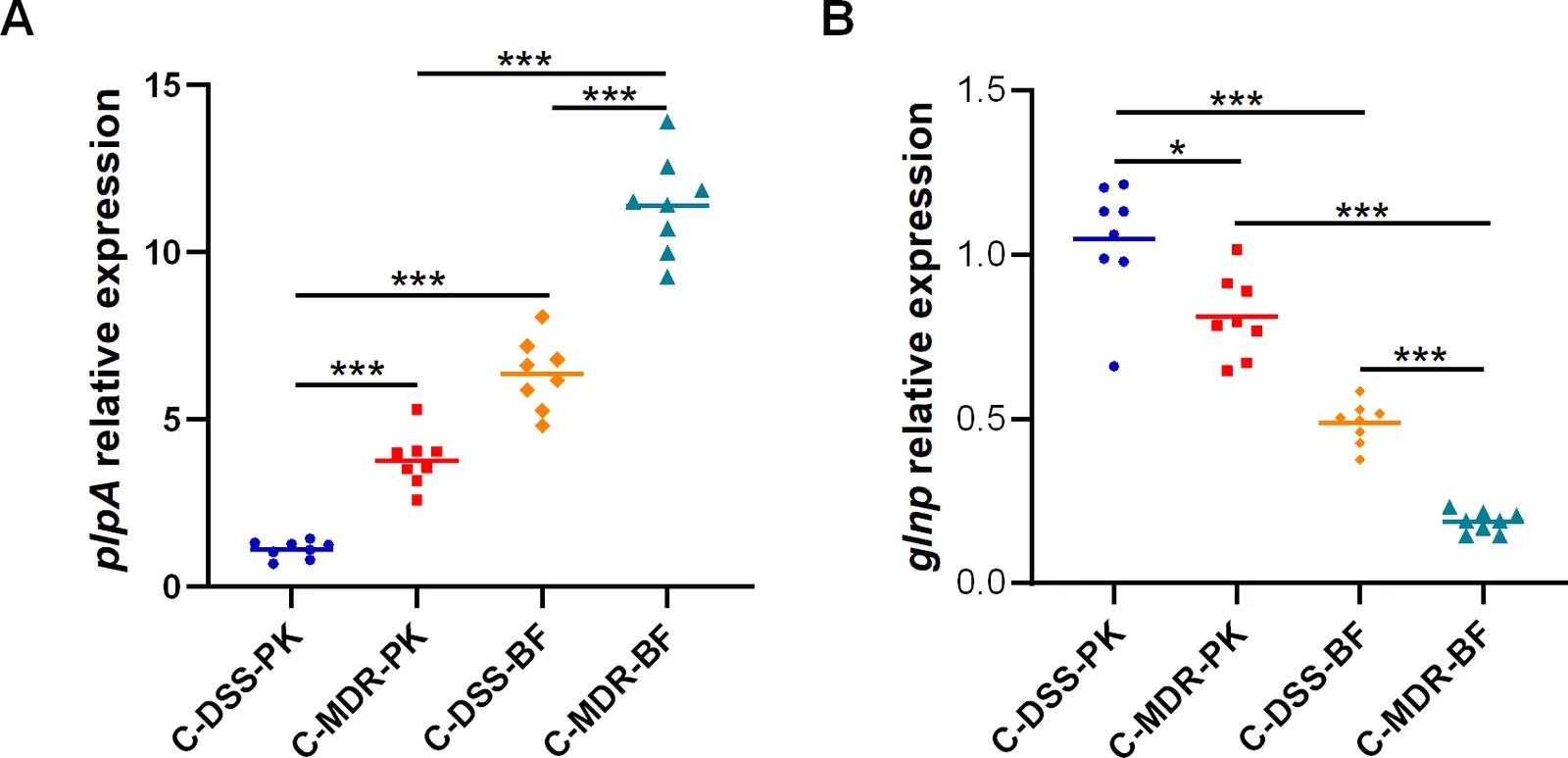
qRT-PCR analysis of *plpA* and *glnP* expression differences in clinically drug-susceptible and multidrug-resistant *H. pylori* strains. Expression levels of *plpA* (**A**) and *glnP* (**B**) were determined in eight C-DSS and eight C-MDR strains under both planktonic (PK) and biofilm (BF) conditions. Results were compared to the values for *plpA* and *glnP* in planktonic WT 26695. The signal was normalized to the *16s rRNA* levels. Data are the means ± standard errors of the means from three independent experiments. Significance was determined by the paired Student’s *t*-test. **, *P* < 0.01; ***, *P* < 0.001.

We determined the expression levels of *glnP* and *plpA* by qRT-PCR in eight clinical multidrug-resistant isolates and eight clinical drug-sensitive isolates from our laboratory collection (42). For both planktonic and biofilm cells, *glnP* expression was significantly lower in multidrug-resistant strains (C-MDR) than in sensitive strains (clinical drug-susceptible strains [C-DSS]), whereas *plpA* showed the opposite trend, with significantly higher expression in multidrug-resistant strains (Fig. 13).

## DISCUSSION

In the present study, we demonstrated that sub-inhibitory concentrations of clinically relevant antibiotics efficiently induce biofilm formation in *H. pylori*. Mechanistic dissection revealed that the two-component system CrdRS orchestrates this process through a dual regulatory mechanism: CrdRS positively activates the outer-membrane ABC transporter substrate-binding protein PlpA to promote exopolysaccharide secretion and matrix assembly, while concurrently repressing the inner-membrane amino acid permease GlnP to relieve transcriptional inhibition of *ansB*, triggering intracellular ROS accumulation and oxidative stress-dependent biofilm maturation. This parallel bidirectional regulation integrates distinct antibiotic stress inputs into a unified biofilm defense program.

Biofilm formation is an active stress-response program triggered by environmental cues, including oxidative stress, nutrient limitation, and antibiotic exposure (16, 20, 21, 24, 25). During *H. pylori* eradication therapy, sub-MIC antibiotic windows arise due to gastric pharmacokinetic constraints (23), converting these drugs into signals that initiate biofilm-mediated defense (24). In selecting antibiotics for this study, we focused on MTZ, AMX, and CIP—agents representing both first-line and rescue regimens for *H. pylori* infection. Clarithromycin, another first-line agent, was excluded because both Krzyżek et al. (26) and our preliminary experiments showed that it fails to induce biofilm formation in *H. pylori*. In our study, sub-MICs of MTZ (1 μg/mL), AMX (1/32 μg/mL), and CIP (1/16 μg/mL) all induced dense biofilms, whereas MIC-level concentrations were bactericidal. Despite targeting distinct cellular processes—DNA synthesis, cell wall synthesis, and DNA gyrase, respectively—all three antibiotics convergently triggered biofilm formation, suggesting a shared sensing mechanism rather than drug-specific pathways. Clinically, high biofilm-forming isolates show significantly higher resistance rates to multiple antibiotics (43), underscoring the importance of maintaining adequate drug levels to avoid sub-MIC windows that may promote treatment failure.

ABC transporters and their substrate-binding proteins (SBPs) play critical roles in nutrient acquisition and solute transport, processes that indirectly support biofilm development (44–46). Functional defects in ABC transport systems have been shown to attenuate biofilm formation in various pathogens (44, 47). In *H. pylori*, PlpA (HP1564) is an SBP of the ABC transporter family (48). Our study revealed that *plpA* is markedly upregulated under sub-inhibitory antibiotic induction, and the *ΔplpA* mutant exhibited significantly reduced polysaccharide content in the extracellular matrix (Fig. 8), leading to structurally compromised biofilms (Fig. 2). Given that the *H. pylori* biofilm EPS is rich in proteomannans and other polysaccharides (49, 50), PlpA may function as a periplasmic SBP that recognizes and delivers substrates required for EPS biosynthesis. The near-complete loss of biofilm integrity in the *ΔplpA* mutant supports a model in which PlpA-mediated substrate transport is a rate-limiting step for matrix assembly during antibiotic-induced biofilm formation, although the precise substrate specificity of PlpA warrants further investigation.

The finding that GlnP, an inner-membrane permease, negatively regulates biofilm formation contrasts with the biofilm-promoting role of PlpA, highlighting functional diversification of ABC transporter components. The *ΔglnP* mutant formed significantly denser and thicker biofilms than WT (Fig. 3), a phenotype confirmed in the clinical H57 background. Transcriptomic analysis revealed marked upregulation of *ansB* (encoding L-asparaginase) in the *ΔglnP* strain. AnsB deaminates L-asparagine to L-aspartate, which enters the TCA cycle via transamination, a process that consumes α-KG (39, 40). We propose that depletion of the α-KG pool may impair the cell’s redox balance by reducing the availability of this metabolite, which itself possesses direct ROS-scavenging activity (41), thereby leading to intracellular ROS accumulation. This model is supported by our experimental data: DCFH-DA staining confirmed elevated ROS in *ΔglnP* biofilm cells, while the *ΔglnPΔansB* double mutant showed WT-level ROS (Fig. 11), and genetic ablation of *ansB* or exogenous catalase reversed the hyper-biofilm phenotype (Fig. 9; Fig. S19 and S20). Low-level ROS act as signaling molecules that promote the planktonic-to-biofilm transition (36). The ROS levels detected in the *ΔglnP* mutant fell within this signaling-competent range, as the mutant exhibited no growth defect (Fig. S5B)—a hallmark of hormetic response, where low-level oxidative stress triggers adaptive protective responses (51). Collectively, these findings suggest a model in which *glnP* downregulation derepresses *ansB*, triggering a metabolic cascade that depletes α-KG, impairs redox balance, and drives ROS-dependent biofilm maturation. Further studies—including direct measurements of intracellular α-KG levels and targeted metabolomic profiling—will be required to fully validate this proposed pathway.

The CrdRS system was originally characterized as a copper-responsive regulator (32) and later shown to respond to nitrosative stress (33). Our study extends this paradigm by identifying CrdRS as a central hub for sub-inhibitory antibiotic-induced biofilm formation via bidirectional regulation of *plpA* and *glnP*. Two lines of evidence support that CrdRS activation under antibiotic stress is independent of secondary ROS: (i) both *plpA* and *glnP* promoters contain paired AC-rich motifs bound by phosphorylated CrdR (Fig. 6 and 7); and (ii) phosphorylation-defective mutants CrdR^D53A^ and CrdS^H173A^ failed to form biofilms under antibiotic induction (Fig. S15 and S16), whereas they retained full biofilm-forming capacity under direct H₂O₂ exposure (Fig. 12). This genetic dissociation demonstrates that CrdRS phosphorylation is required for antibiotic signal transduction but dispensable for ROS-induced biofilm formation. Whether CrdS directly binds these structurally diverse antibiotics or senses a common downstream consequence—such as membrane stress or proton motive force dissipation—remains to be determined; future biochemical and structural studies are required to distinguish among these possibilities.

**Fig 14:**
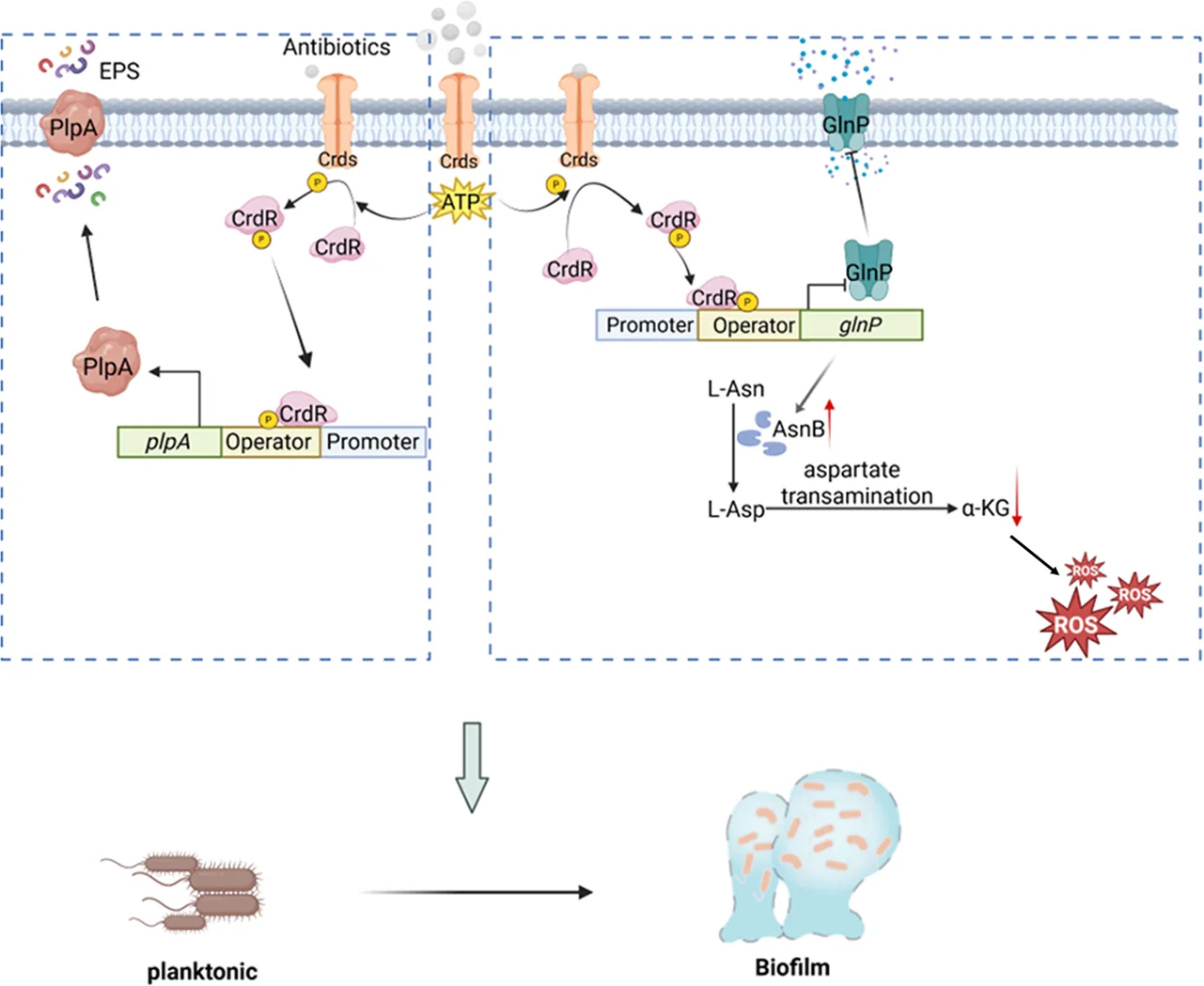
Schematic model of CrdRS-mediated regulation of sub-inhibitory antibiotic-induced biofilm formation in *H. pylori*. Upon antibiotic exposure, the histidine kinase CrdS autophosphorylates and transfers the phosphoryl group to the response regulator CrdR. Phosphorylated CrdR directly activates *plpA* transcription to promote exopolysaccharide secretion and matrix assembly (left), while concurrently repressing *glnP* transcription, leading to intracellular ROS accumulation that drives biofilm maturation (right). This dual regulatory mechanism coordinates antibiotic-induced biofilm development.

In summary, this study establishes a comprehensive model (Fig. 14) in which the CrdRS two-component system serves as a central antibiotic-sensing hub that orchestrates biofilm formation in *H. pylori* through bidirectional regulation of ABC transporters, integrating extracellular matrix assembly and intracellular redox signaling into a coordinated defensive response. Importantly, the differential expression of *plpA* and *glnP* observed in clinical multidrug-resistant versus drug-susceptible isolates (Fig. 13), together with the reduced biofilm formation observed in H57 *ΔplpA* and *ΔglnP* mutants (Fig. S8 and S11), links this CrdRS-PlpA/GlnP regulatory axis to clinically relevant antibiotic resistance. These findings identify CrdRS as a promising therapeutic target for disrupting biofilm-mediated drug tolerance and improving treatment outcomes in multidrug-resistant *H. pylori* infections.

## MATERIALS AND METHODS

### Bacterial strains and culture conditions

*H. pylori* reference strain 26695 and clinical isolates (obtained from a gastric ulcer patient with three failed standard triple therapy courses at Qiannan Prefecture People’s Hospital, Guizhou, China) were used in this study. The resistance profiles of these clinical isolates have been previously characterized (42); among them, eight C-MDR and eight C-DSS isolates were selected for this study based on their resistance patterns. *H. pylori* was routinely cultured on Columbia agar supplemented with 5% (vol/vol) defibrinated sheep blood and 1% (vol/vol) antibiotic cocktail (polymyxin, trimethoprim, amphotericin B, vancomycin) under microaerobic conditions (5% O₂, 10% CO₂, 85% N₂) at 37°C. Liquid cultures were grown in Brucella broth containing 10% (vol/vol) newborn calf serum with shaking at 120 rpm in sealed gas jars. Stocks were stored in brain heart infusion with 30% (vol/vol) glycerol at –80°C. *Escherichia coli* TOP10 and BL21(DE3) were used for cloning and protein expression, respectively, and were cultured in Luria–Bertani medium at 37°C with shaking at 200 rpm. Deletion and complementation mutants were maintained with selected antibiotics: kanamycin (30 μg/mL) or chloramphenicol (100 μg/mL). All antibiotics were purchased from Sigma-Aldrich.

### Construction of *H. pylori* gene deletion, complementation, and site-directed mutants

Gene deletion mutants were constructed in the *H. pylori* 26695 background by allelic replacement with the *aphA*-3 kanamycin resistance cassette (52). Complementation strains were generated by inserting the target gene with its native promoter into the intergenic region between *hp0203* and *hp0204* using plasmid pBHKP252 (53), followed by natural transformation and allelic exchange. The clinical isolate H57 deletion mutants (*ΔplpA*, *ΔglnP*) were obtained using the same strategy. Phosphorylation-site mutants (CrdR^D53A^, CrdS^H173A^) were constructed by a multi-step PCR method with specific inner mutagenic primers and high-fidelity DNA polymerase. All mutants were verified by PCR and sequencing (54). Primer sequences are listed in Table S1.

### Growth curve determination

Overnight *H. pylori* cultures were adjusted to an initial OD₆₀₀ of 0.08 and inoculated into Brucella broth supplemented with 10% heat-inactivated newborn calf serum. Cultures were incubated microaerobically at 37°C with shaking at 120 rpm. OD₆₀₀ was measured every 12 h for 144 h. Data are expressed as mean ± SD from three biological replicates.

### Biofilm formation

Biofilms were cultivated using the colony biofilm assay (52). Logarithmic-phase cultures were adjusted to 1 × 10⁸ CFU/mL, and 25 μL aliquots were spotted onto nitrocellulose membranes (1 cm²) placed on solid medium. After antibiotic concentration-gradient screening, optimal sub-inhibitory induction conditions were determined as MTZ (1 μg/mL), AMX (1/32 μg/mL), and CIP (1/16 μg/mL). All subsequent experiments used these optimal concentrations; 50 μM H₂O₂ served as the positive control. Plates were incubated inverted under microaerobic conditions at 37°C for 2–3 days. Mature biofilms were imaged using a Zeiss stereo microscope.

### Confocal laser scanning microscopy

Mature biofilms were gently washed with PBS to remove planktonic cells and stained with SYTO 9/propidium iodide (LIVE/DEAD Cell Viability Assay) for bacterial viability, Calcofluor White for exopolysaccharides, or SYPRO Ruby Biofilm Matrix Stain for extracellular proteins. Staining was performed in the dark at 37°C for 30 min. After washing, samples were visualized and three-dimensionally reconstructed using a Zeiss LSM 980 confocal laser scanning microscope.

### Scanning electron microscopy

Biofilms were rinsed with sterile PBS and fixed with 2.5% glutaraldehyde at 4°C overnight. After washing with 0.1 M phosphate buffer (pH 7.4), samples were post-fixed in 1% osmium tetroxide for 1–2 h at room temperature, dehydrated through a graded ethanol series (30%–100%), freeze-dried, sputter-coated with gold, and examined under a Hitachi SU8100 scanning electron microscope.

### Crystal violet biofilm quantification

Triplicate mature biofilms were gently washed and eluted from membranes with 1 mL PBS. One milliliter of 1% crystal violet solution was added, mixed, and incubated at room temperature for 25 min. After washing with PBS to remove excess dye, bound crystal violet was solubilized with 1 mL absolute ethanol, and OD₅₉₅ was measured using an Agilent SH1M2F Multimode Reader. Absolute ethanol served as the blank. Three biological replicates were performed.

### RNA extraction and quantitative real-time PCR

Total RNA was extracted from *H. pylori* cells (OD₆₀₀ = 1.0) using the TRIzol method. Genomic DNA was eliminated, and reverse transcription was performed with the Evo M-MLV RT Mix Kit with gDNA Clean. qRT-PCR was conducted using ChamQ SYBR Color qPCR Master Mix on a CFX96 system. The 16S rRNA gene was used as the internal reference, and relative expression was calculated by the 2^−ΔΔCT^ method (55). Semi-quantitative RT-PCR products were resolved by agarose gel electrophoresis. Primer sequences are provided in Table S1.

### Luciferase reporter gene assay

The promoter regions of *glnP* and *plpA* were cloned into the pGL3-Basic vector to generate pGL3-OP*_glnP_* and pGL3-OP*_plpA_*. These reporter plasmids were co-transformed with pET32a-*crdR* into *E. coli* BL21(DE3) in various combinations. Luciferase activity was measured using a Luciferase Reporter Gene Assay Kit and a Centro XS3 LB960 luminometer. Empty pGL3-Basic and pET32a vectors served as controls. Experiments were performed in triplicate.

### Protein expression and purification

Wild-type *crdR* and mutant *crdR*^D53A^ (stop codon removed) were cloned into pET-32a and transformed into *E. coli* BL21(DE3). Strains were grown to OD₆₀₀ ≈ 0.6–0.8 at 37°C, induced with 0.5 mM IPTG, and incubated at 16°C with shaking at 200 rpm for 12–16 h. Recombinant proteins were purified by Ni-IDA Sepharose affinity chromatography with imidazole gradient elution. Purity was assessed by 10% SDS-PAGE and Coomassie brilliant blue staining.

### *In vitro* protein phosphorylation and electrophoretic mobility shift assay

Purified CrdR protein was subjected to *in vitro* chemical phosphorylation using lithium potassium acetyl phosphate as the phosphate donor. The reaction mixture contained 50 mM Tris-HCl (pH 8.0), 5 mM MgCl₂, 50 mM KCl, 1 mM dithiothreitol, and 50 mM lithium potassium acetyl phosphate. The reaction was incubated at 25°C for 30 min in a metal bath, as described previously (56).

EMSA was performed as previously described (54). The *crdA* promoter (OP*_crdA_*) served as a positive control (32) and the 16S rRNA gene promoter (OP*_16S_*) as a negative control. Defined DNA probes encompassing conserved promoter regions were PCR-amplified (for probe details, see Fig. 6E and 7E). Purified CrdR protein was subjected to *in vitro* phosphorylation and incubated with individual probes in binding buffer for 30 min. Binding reactions were resolved on 6% non-denaturing polyacrylamide gels in 0.25× TBE buffer (1% glycerol) at 180 V for 60 min. Phosphorylation site mutant CrdR^D53A^ was processed identically. Gels were stained with GelRed and imaged under UV transillumination.

### Reactive oxygen species detection

*H. pylori* biofilms cultured for 3 days under sub-inhibitory antibiotics were stained with 10 μM DCFH-DA in PBS at 37°C for 30 min in the dark. After washing, intracellular ROS fluorescence was detected using a Beckman Coulter CytoFLEX flow cytometer and a Zeiss LSM 980 confocal microscope (excitation 488 nm, emission 525 nm). Mean fluorescence intensity was quantified using ImageJ. All experiments were performed with at least three independent biological replicates.

### RNA sequencing and bioinformatic analysis

Biofilm cells of *H. pylori* 26695 and *ΔglnP* mutant strains were harvested after 72 h of induction with sub-inhibitory antibiotics (MTZ 1 μg/mL, AMX 1/32 μg/mL, or CIP 1/16 μg/mL) using the colony biofilm assay described above. Total RNA was extracted and subjected to RNA-seq by Shanghai Majorbio Bio-Pharm Technology Co., Ltd. (China). RNA-seq was performed on samples from the three antibiotic treatment conditions as independent biological conditions without technical replicates. To ensure robustness, genes showing consistent differential expression across all three antibiotic treatments (fold change ≥ 2, *P* < 0.05) were prioritized for further analysis.

### Statistical analysis

Data are presented as mean ± SEM from at least three independent biological replicates. Pairwise comparisons were performed using Student’s *t*-test; multiple group comparisons were analyzed by one-way ANOVA. *P* < 0.05 was considered statistically significant. GraphPad Prism version 10.6.0 was used for all statistical analyses and graphical representation.

## Data Availability

The RNA-seq data generated in this study have been deposited in the NCBI Gene Expression Omnibus (GEO) under accession numbers PRJNA1108659 and PRJNA1473680. All other data supporting the findings of this study are available within the manuscript and its Supplemental material.

## SUPPLEMENTAL MATERIAL

Supplemental material aac.00882-26-s0001.pdf Fig. S1 to S21; Table S1.

ASM does not own the copyrights to Supplemental Material that may be linked to, or accessed through, an article. The authors have granted ASM a non-exclusive, world-wide license to publish the Supplemental Material files. Please contact the corresponding author directly for reuse.
