# Supplementary material for: The two-component system CrdRS mediates sub-inhibitory antibiotic-induced biofilm formation in Helicobacter pylori via bidirectional regulation of transporters PlpA and GlnP
Source: Antimicrob Agents Chemother. 2026 Oct 5;70(11):e00882-26. doi: 10.1128/aac.00882-26 (PMC13731178; doi:10.1128/aac.00882-26)
Supplement: Supplementary file 1 [file aac.00882-26-s0001.pdf]

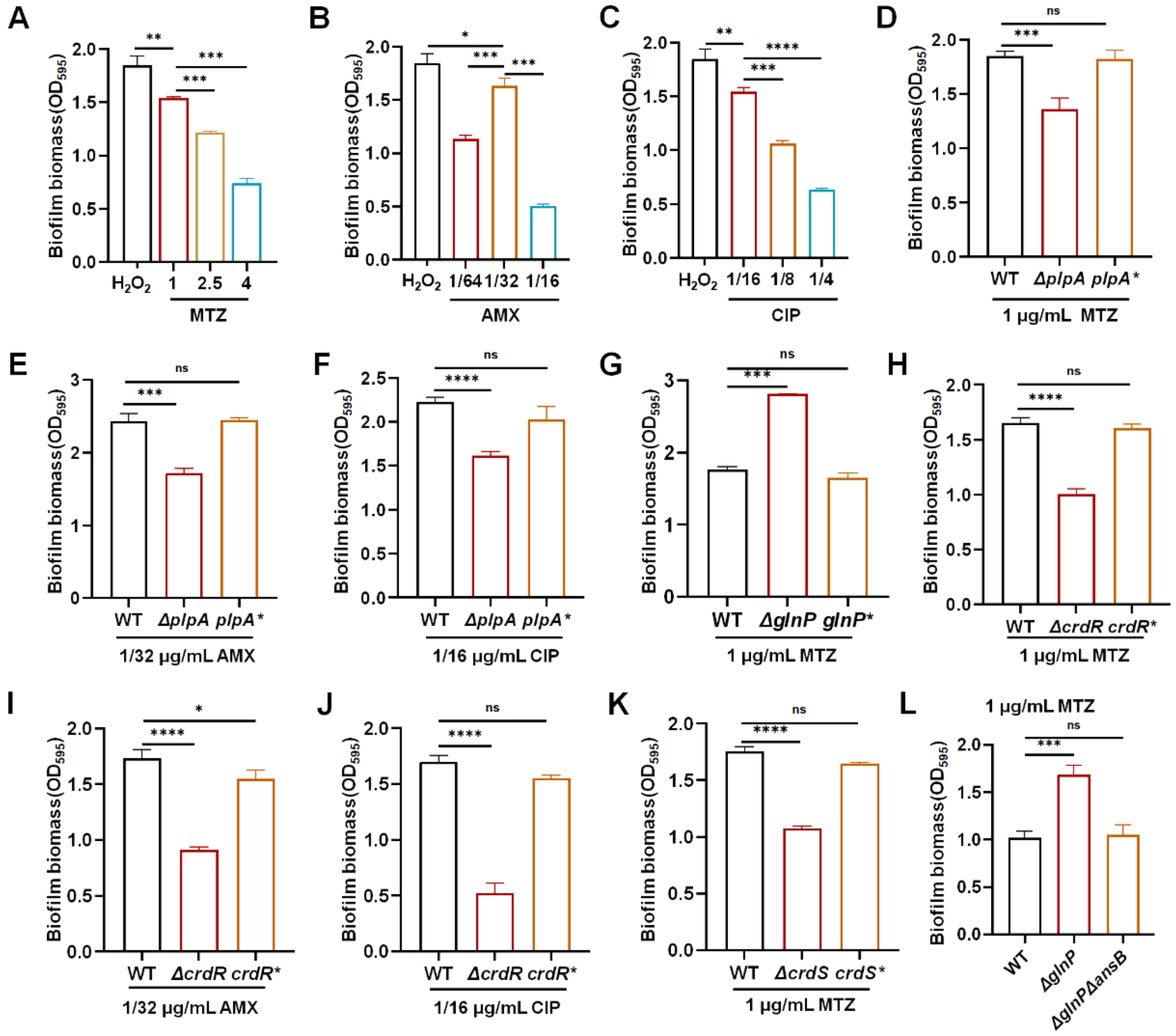

Fig S1 Quantification of biofilm biomass in *H. pylori* strains treated with sub-inhibitory antibiotics. (A–C) Biofilm biomass (OD_{595}) of wild-type (WT) strain 26695 treated with 50 μM H_2O_2 (positive control) and a range of concentrations of metronidazole (MTZ), amoxicillin (AMX), or ciprofloxacin (CIP). (D–L) Biofilm biomass of gene deletion mutants (*ΔplpA*, *ΔglnP*, *ΔcrdR*, and *ΔcrdS*), complemented strains (*plpA**, *glnP**, *crdR**, and *crdS**), and the *ΔglnPΔansB* double mutant following induction with optimal sub-inhibitory concentrations of MTZ (1 μg/mL), AMX (1/32 μg/mL), or CIP (1/16 μg/mL). Biofilm biomass was quantified by crystal violet staining. Data are presented as mean ± SD from three independent biological replicates. Statistical significance: ns, not significant; *, $P < 0.05$; **, $P < 0.01$; ***, $P < 0.001$; ****, $P < 0.0001$.

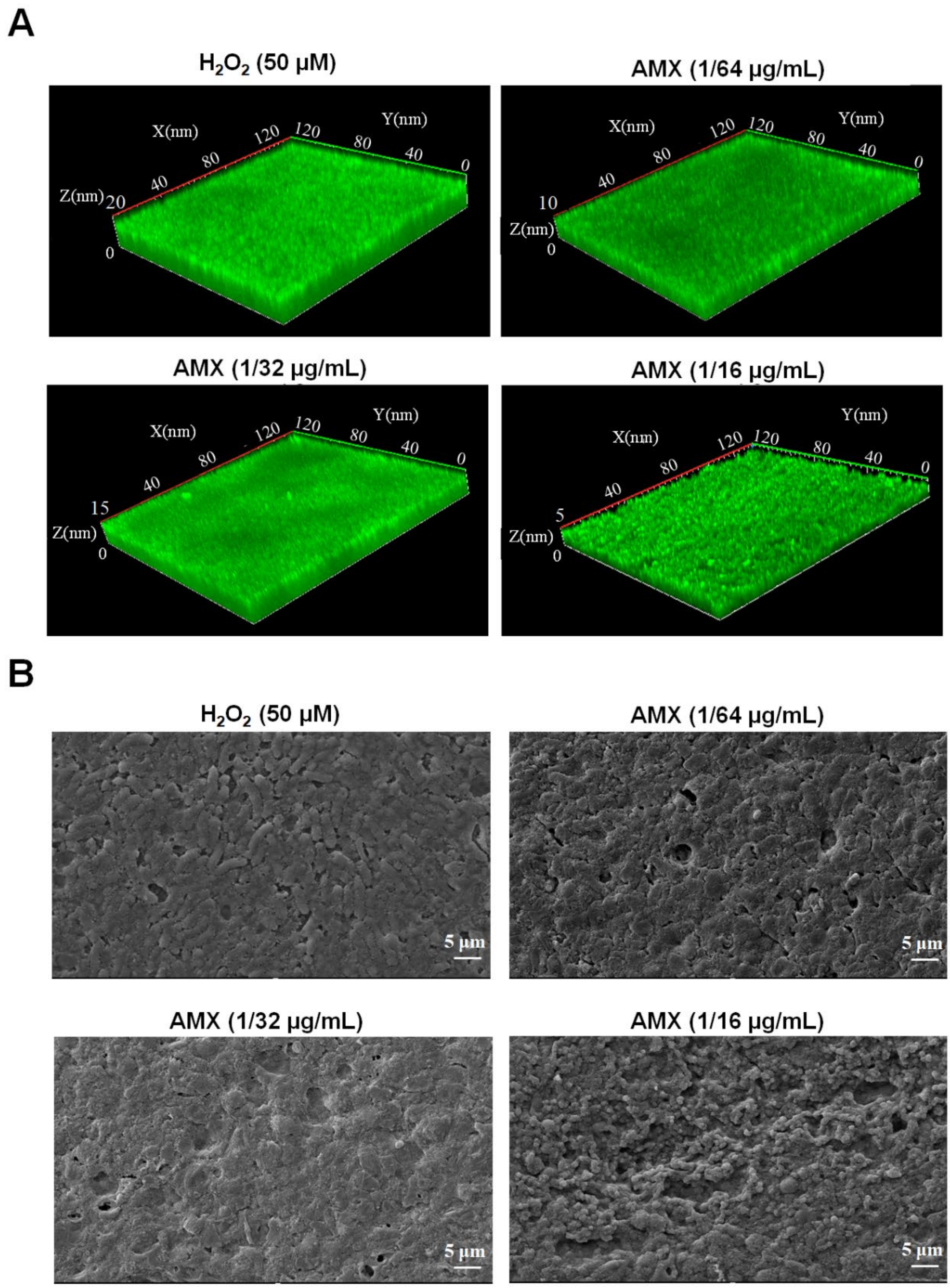

Fig S2. Sub-inhibitory concentrations of AMX induce *H. pylori* biofilm formation. Mature biofilms were cultivated and analyzed following the methodology described for Figure 1. (A) Representative confocal laser scanning microscopy (CLSM) images of biofilms stained with SYTO™ 9 (green) using the LIVE/DEAD Cell Viability Assay. (B) Representative scanning electron microscopy (SEM) images of biofilm ultrastructure. Scale bar, 5 µm. Data are representative of three independent experiments. Note: This experimental group was conducted in parallel with the same batch as the experiment presented in Fig. 1; the shared control group images are

identical to those in Figure 1.

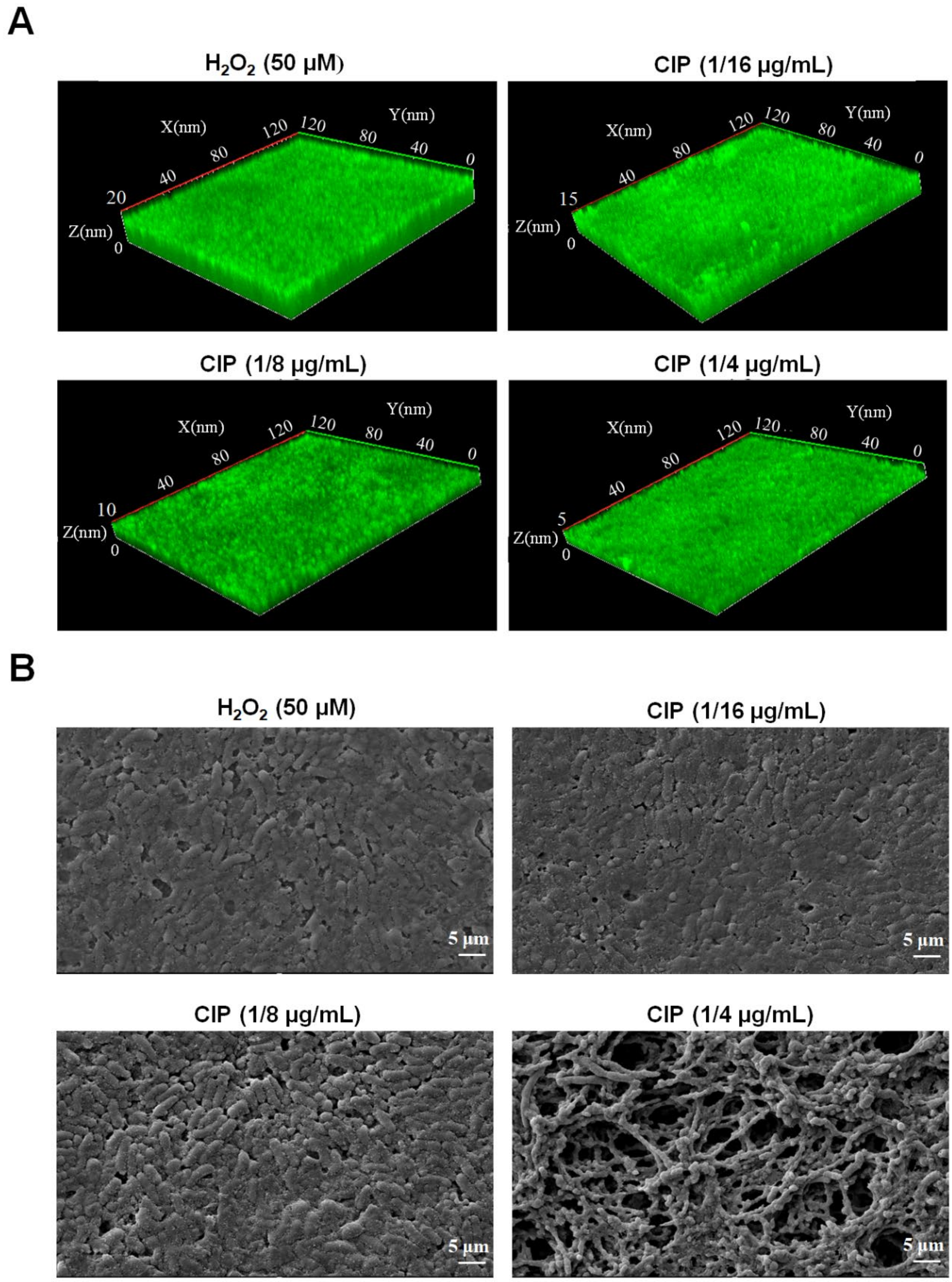

Fig S3 Sub-inhibitory concentrations of CIP induce *H. pylori* biofilm formation. Mature biofilms were cultivated and analyzed following the methodology described for Fig 1. (A) Representative CLSM images of biofilms stained with SYTO™ 9 (green) using the LIVE/DEAD Cell Viability Assay. (B) Representative SEM images of biofilm ultrastructure. Scale bar, 5 μm. Data are representative of three independent experiments. Note: This experimental group was conducted in parallel with the same

batch as the experiment presented in Figure 1; the shared control group images are identical to those in Figure 1.

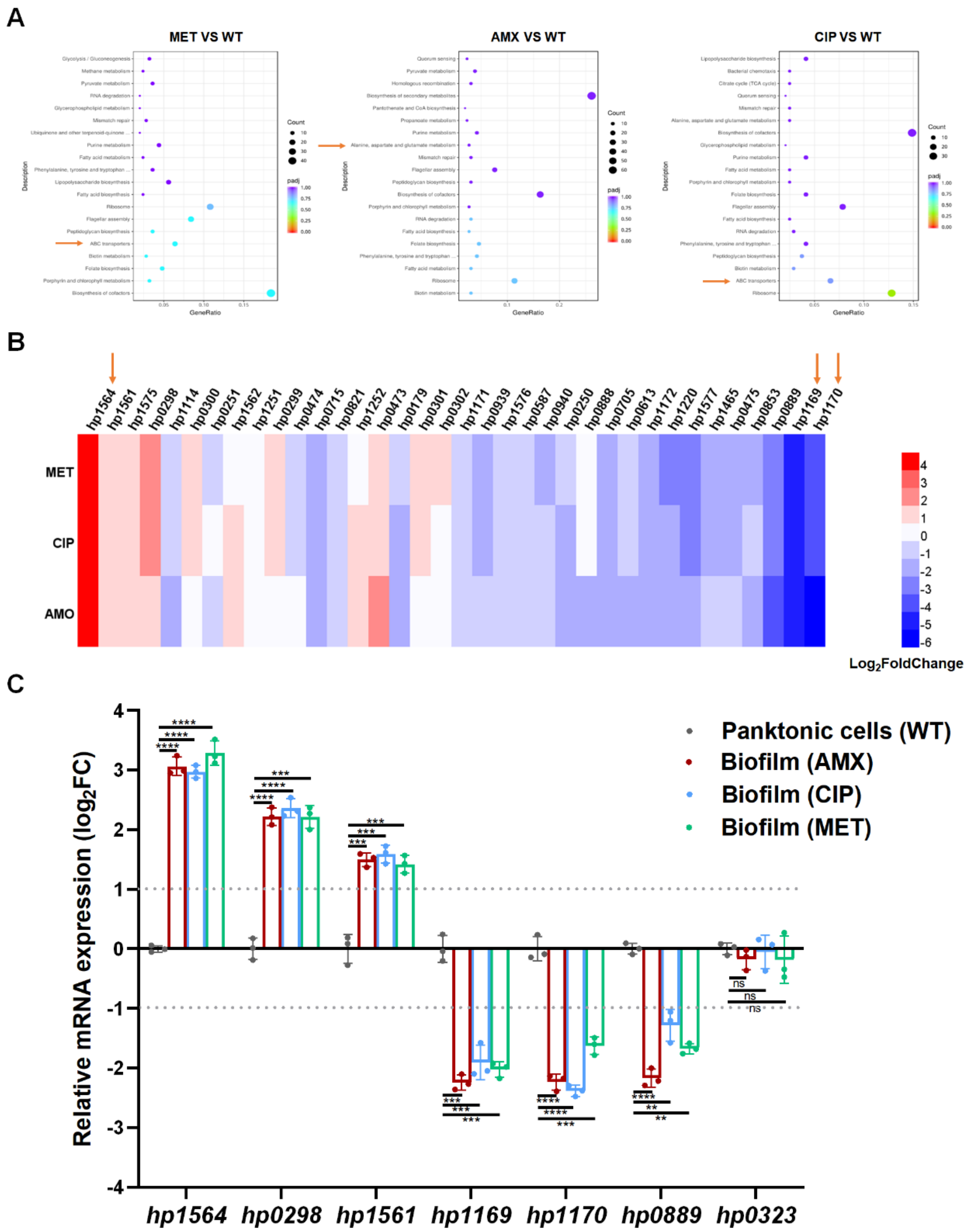

Fig S4. Differential expression of ABC transporter genes in *H. pylori* biofilm cells induced by sub-inhibitory antibiotics. (A) KEGG pathway enrichment bubble plots (from left to right: MTZ vs WT, AMX vs WT, and CIP vs WT, respectively). The x-axis represents gene ratio, the y-axis represents pathway description, bubble size indicates gene count, and color denotes enrichment significance (padj value). (B) Heatmap showing expression patterns of key ABC transporter genes under the three antibiotic treatments. Red indicates upregulation; blue indicates downregulation. Arrows indicate *plpA* (*hp1564*) and the *glnP* genes (*hp1169* and *hp1170*).and the *glnP* genes (*hp1169*

and *hp1170*). (C) qRT-PCR validation of representative genes selected from the heatmap in (B), including significantly upregulated genes (*hp1564*, *hp0298*, *hp*1561), significantly downregulated genes (*hp1169*, *hp1170*, *hp0889*), and non-differentially expressed genes *(hp0323*). Relative mRNA expression ($\log_2$ fold change) in biofilm cells induced by sub-inhibitory AMX, CIP, or MTZ is shown, normalized to untreated planktonic WT cells. Data are presented as mean ± SD from three independent biological replicates, each with three technical replicates. Statistical significance: ns, not significant; *, $P < 0.05$; **, $P < 0.01$; ***, $P < 0.001$; ****, $P < 0.0001$.

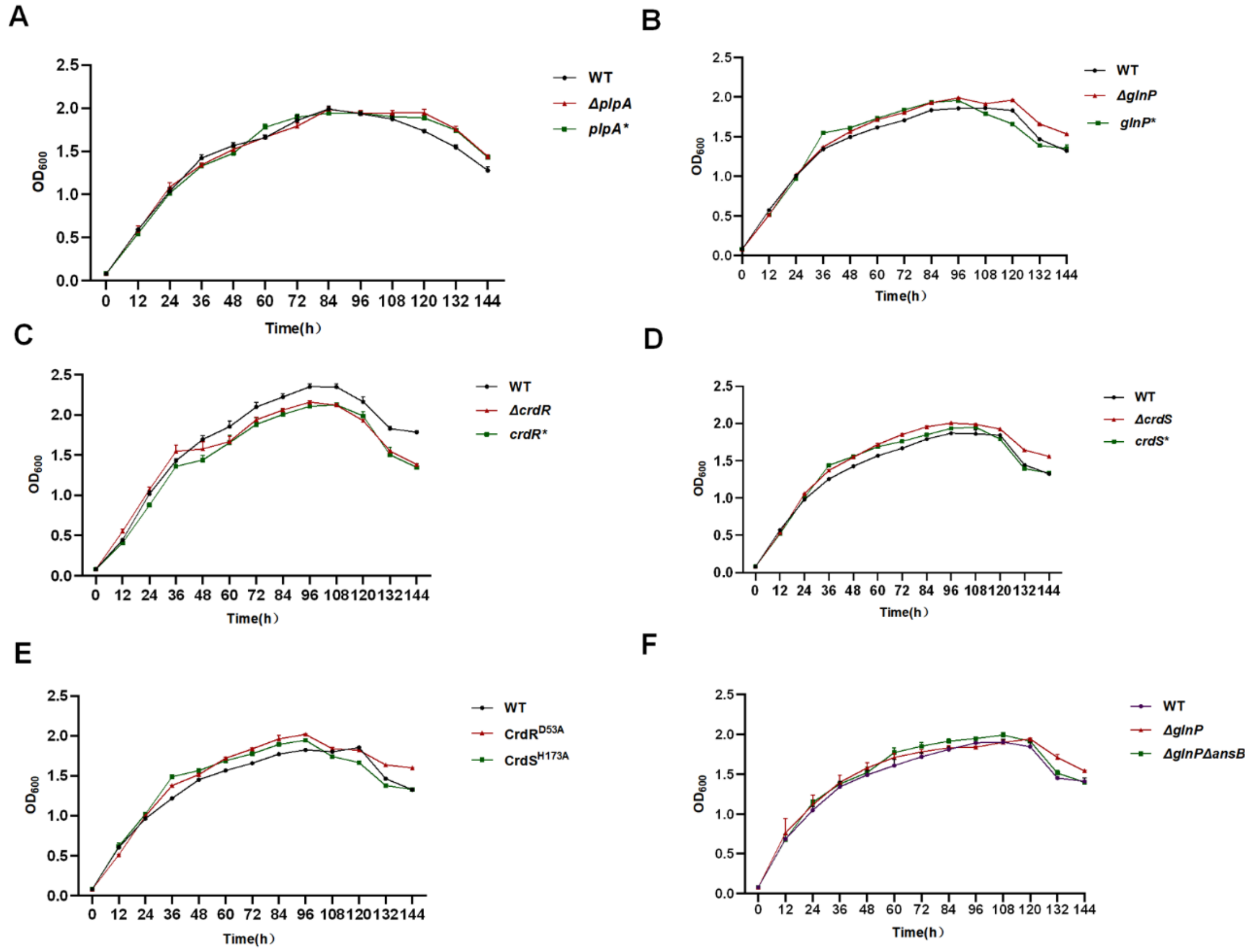

Fig S5 Growth curves of WT, deletion, complementation, and site-directed mutant strains. (A) WT, *ΔglnP*, and *glnP**; (B) WT, *ΔplpA*, and *plpA**; (C) WT, *ΔcrdR*, and *crdR**; (D) WT, *ΔcrdS,* and *crdS**; (E) WT, CrdR^{D53A}, and CrdS^{H173A}; (F) WT, *ΔglnP*, and *ΔglnPΔansB*. Strains were inoculated into *H. pylori* liquid medium at an initial OD_{600} of 0.08 and incubated at 37°C with shaking at 120 rpm. OD_{600} was measured at 12 h intervals. Data are presented as the mean of three biological replicates.

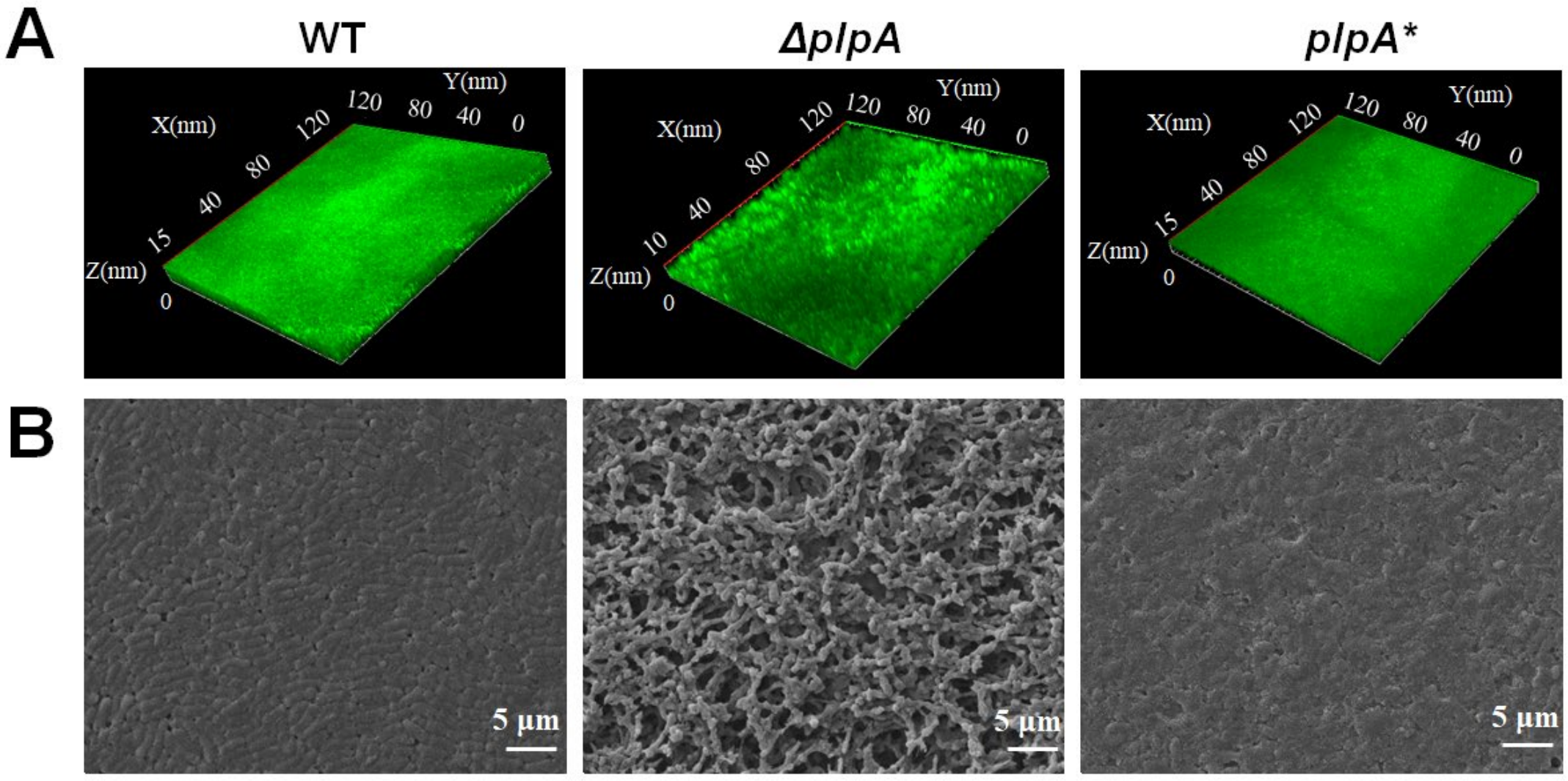

Fig S6 Sub-inhibitory AMX-induced biofilm formation is impaired in the *ΔplpA* mutant. WT, *ΔplpA*, and *plpA** strains were cultured on medium containing AMX (1/32 μg/mL) for 3 days to allow mature biofilm development. (A) Representative CLSM images of biofilms stained with SYTO™ 9 (green) using the LIVE/DEAD Cell Viability Assay. (B) Representative SEM images of biofilm ultrastructure. Scale bar, 5 μm. Data are representative of three independent experiments.

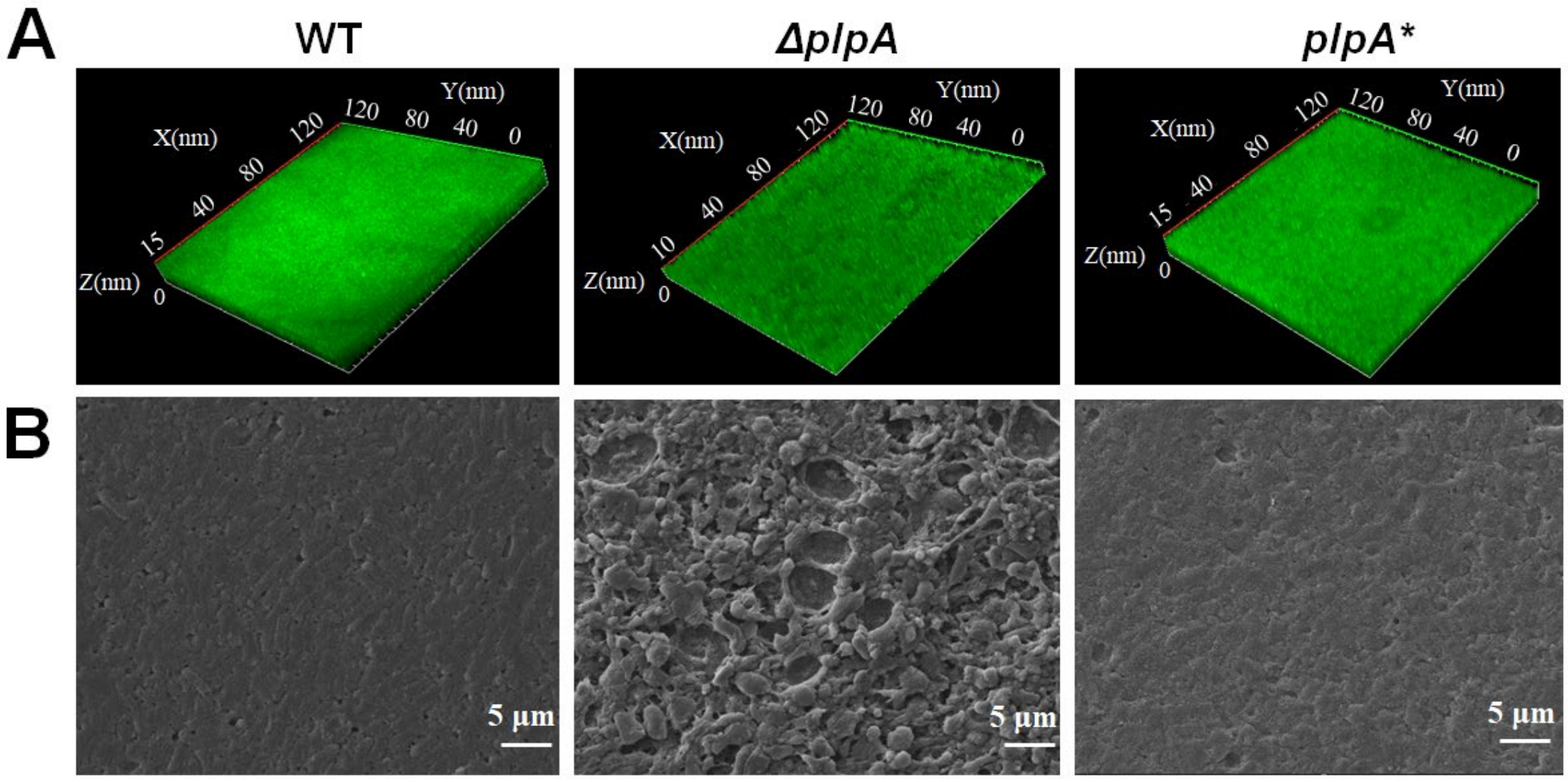

Fig S7 Sub-inhibitory CIP-induced biofilm formation is impaired in the *ΔplpA* mutant. WT, *ΔplpA*, and *plpA** strains were cultured on medium containing CIP (1/16 μg/mL) for 3 days to allow mature biofilm development. (A) Representative CLSM images of biofilms stained with SYTO™ 9 (green) using the LIVE/DEAD Cell Viability Assay. (B) Representative SEM images of biofilm ultrastructure. Scale bar, 5 μm. Data are representative of three independent experiments.

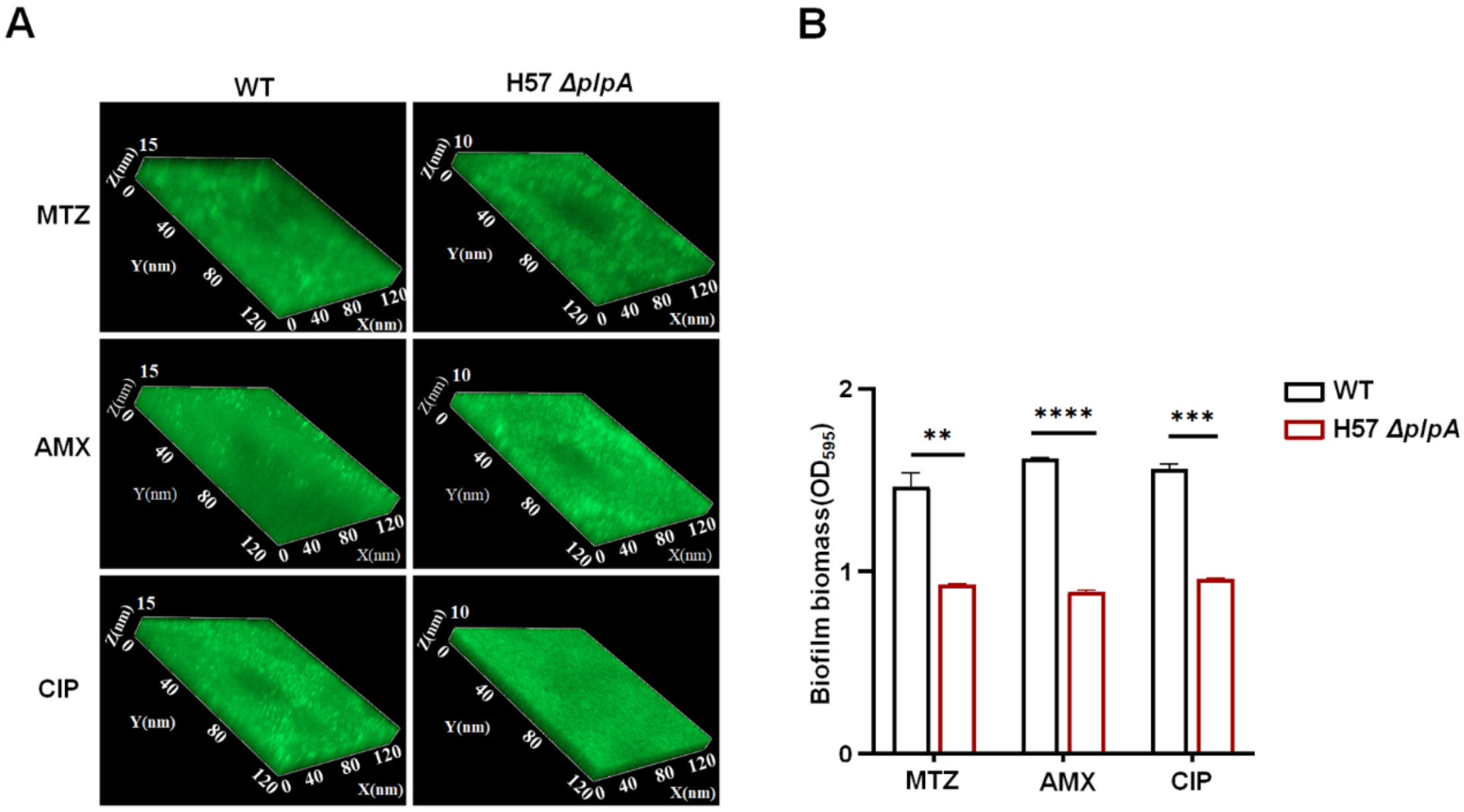

Fig S8 Deletion of *plpA* attenuates biofilm formation in the clinical isolate H57 under sub-inhibitory antibiotic induction. Biofilms were induced using three sub-inhibitory antibiotic concentrations: MTZ (1 μg/mL), AMX (1/32 μg/mL), and CIP (1/16 μg/mL). (A) Three-dimensional CLSM reconstructions showing biofilm architecture of H57 wild-type (WT) and H57Δ*plpA* strains. (B) Biofilm biomass quantified by crystal violet staining (OD_{595}). Data are presented as mean ± SD from three independent experiments. Statistical significance: **, $P < 0.01$; ***, $P < 0.001$; ****, $P < 0.0001$.

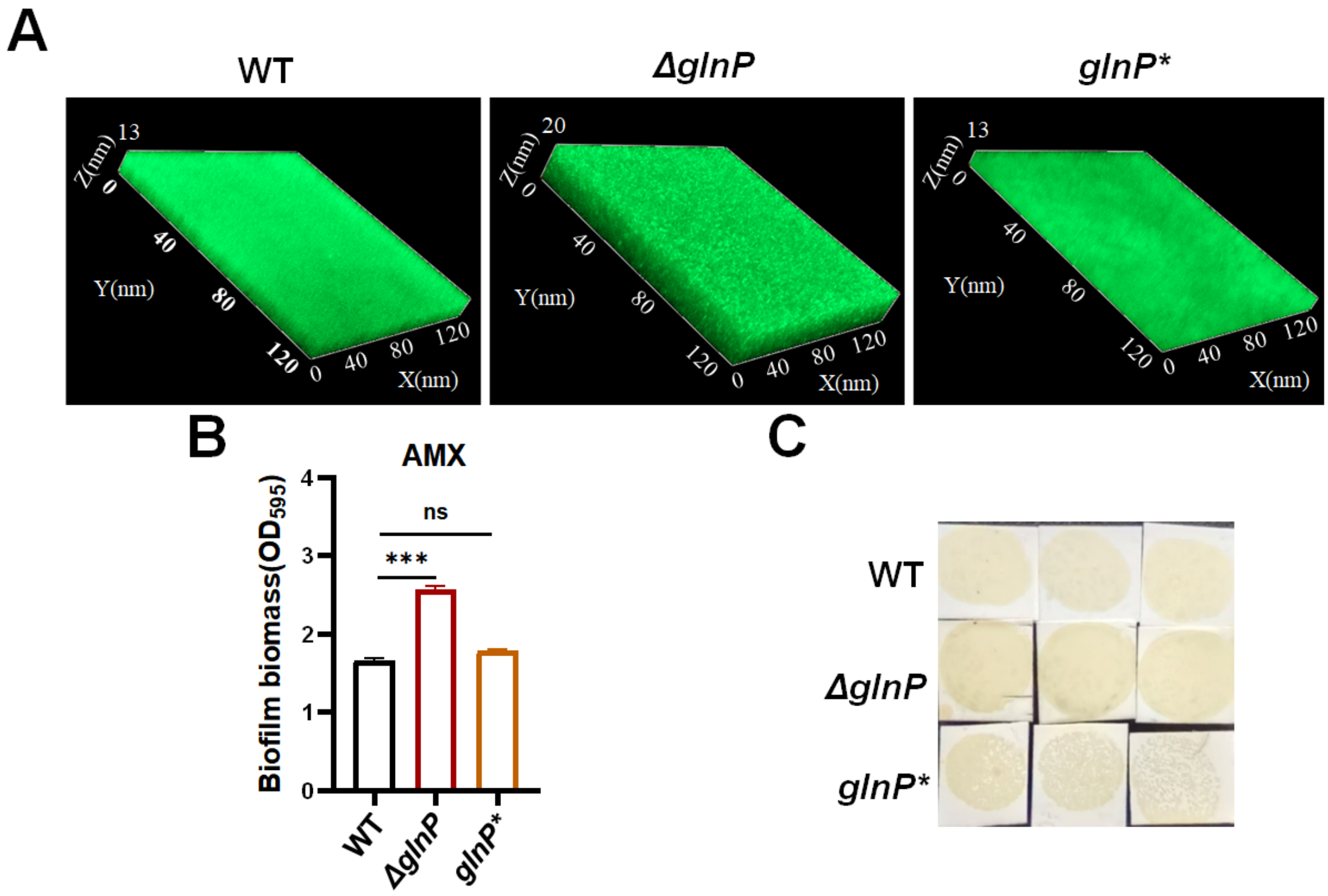

Fig S9 Deletion of *glnP* enhances sub-inhibitory AMX-induced biofilm formation in *H. pylori*. WT, *ΔglnP*, and *glnP** strains were cultured on medium containing AMX (1/32 μg/mL) for 3 days. (A) Representative three-dimensional CLSM images of

biofilms stained with SYTO™ 9 (green). (B) Biofilm biomass quantified by crystal violet staining (OD_{595}). (C) Representative images of biofilms formed on nitrocellulose membranes. Data are presented as mean ± SD from three independent experiments. Statistical significance: ns, not significant; ***, $P < 0.001$.

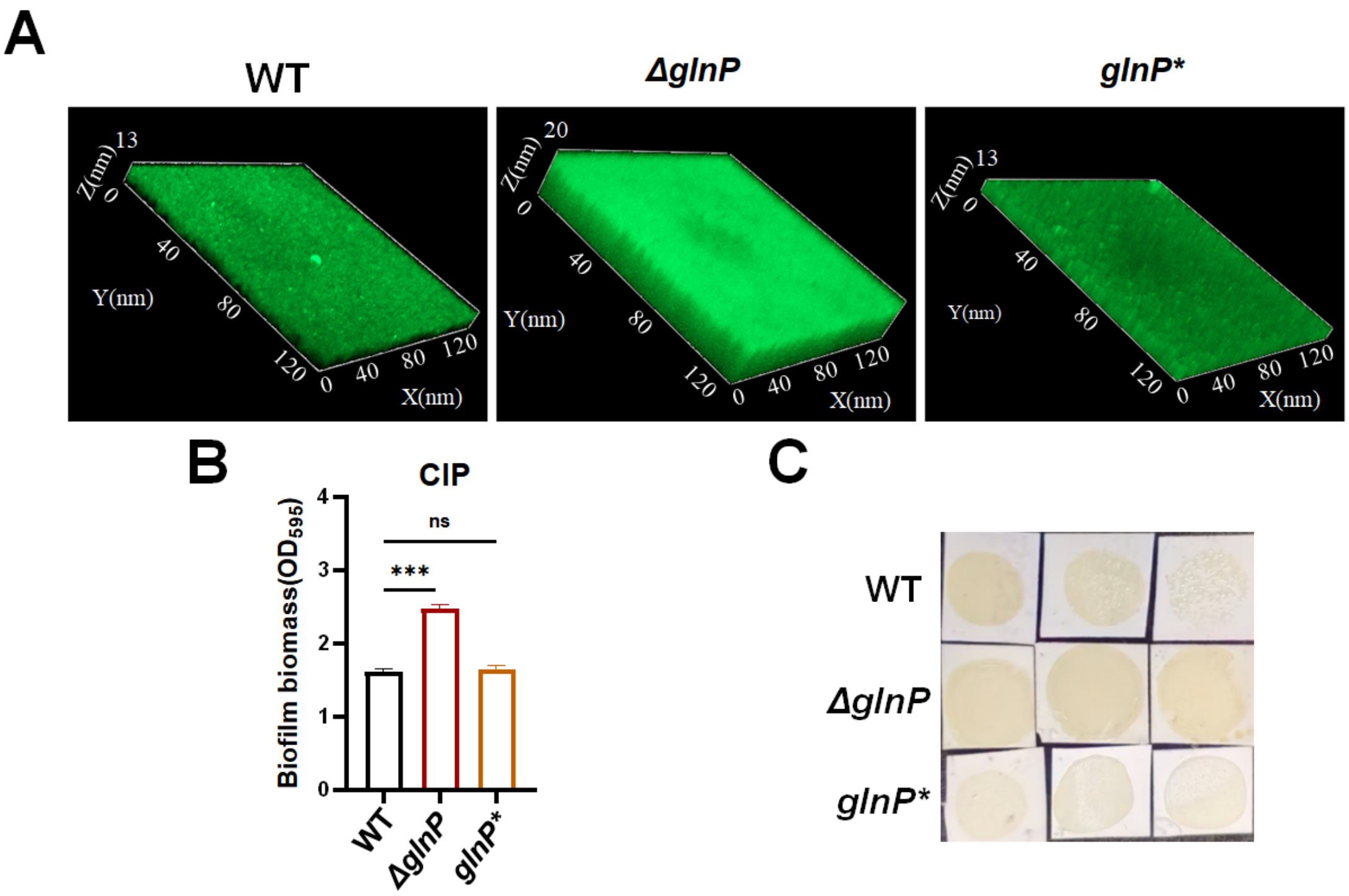

Fig S10 Deletion of *glnP* enhances sub-inhibitory CIP-induced biofilm formation in *H. pylori*. WT, *ΔglnP*, and *glnP** strains were cultured on medium containing CIP (1/16 μg/mL) for 3 days. (A) Representative three-dimensional CLSM images of biofilms stained with SYTO™ 9 (green). (B) Biofilm biomass quantified by crystal violet staining (OD_{595}). (C) Representative images of biofilms formed on nitrocellulose membranes. Data are presented as mean ± SD from three independent experiments. Statistical significance: ns, not significant; ***, $P < 0.001$.

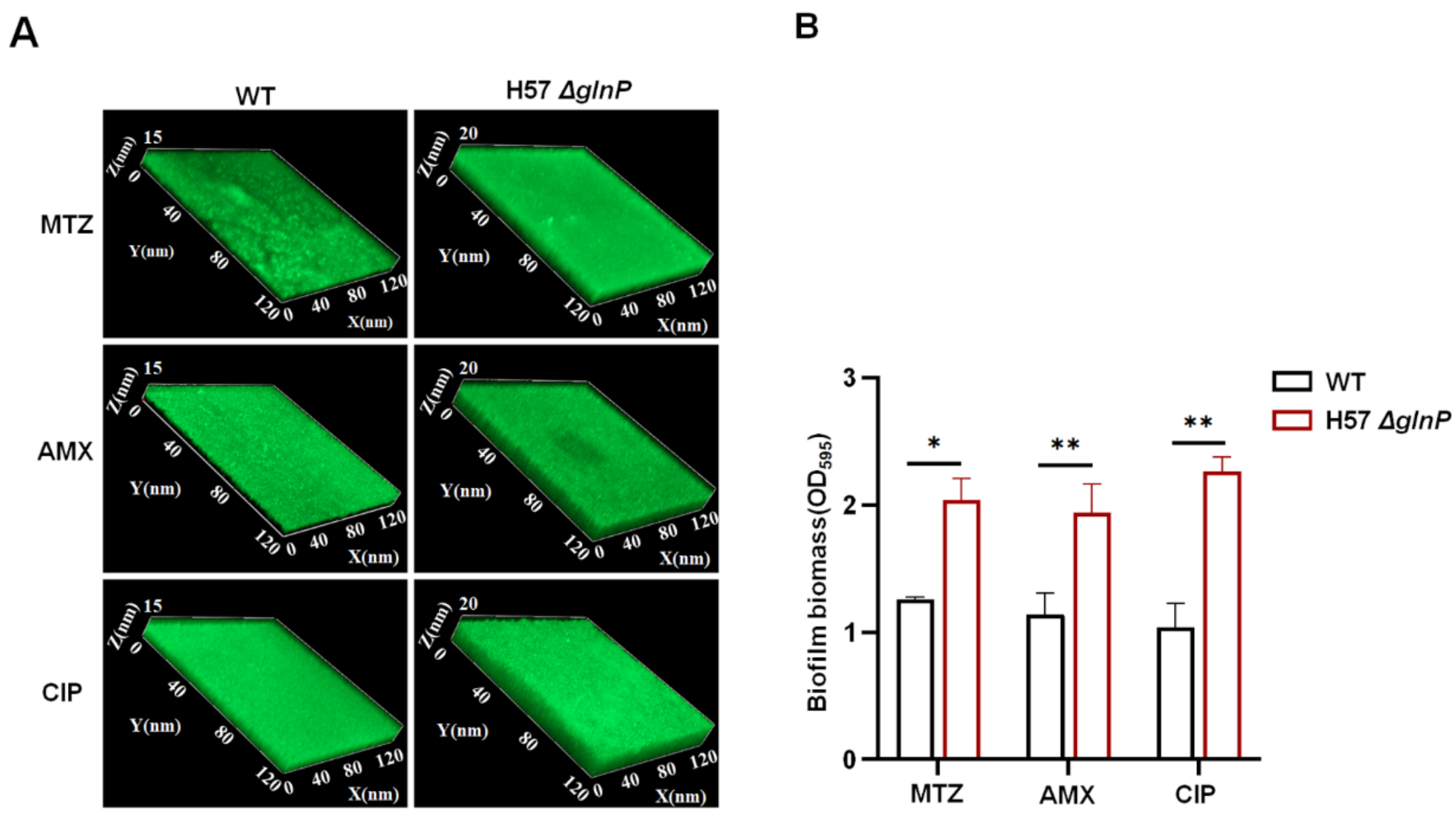

Fig S11. Deletion of *glnP* enhances biofilm formation in the clinical isolate H57 under sub-inhibitory antibiotic induction. Biofilms were induced using three sub-inhibitory antibiotic concentrations: MTZ (1 μg/mL), AMX (1/32 μg/mL), and CIP (1/16 μg/mL). (A) Three-dimensional CLSM reconstructions showing biofilm architecture of H57 wild-type (WT) and H57*ΔglnP* strains. (B) Biofilm biomass quantified by crystal violet staining (OD_{595}). Data are presented as mean ± SD from three independent experiments. Statistical significance: *, $P < 0.05$; **, $P < 0.01$.

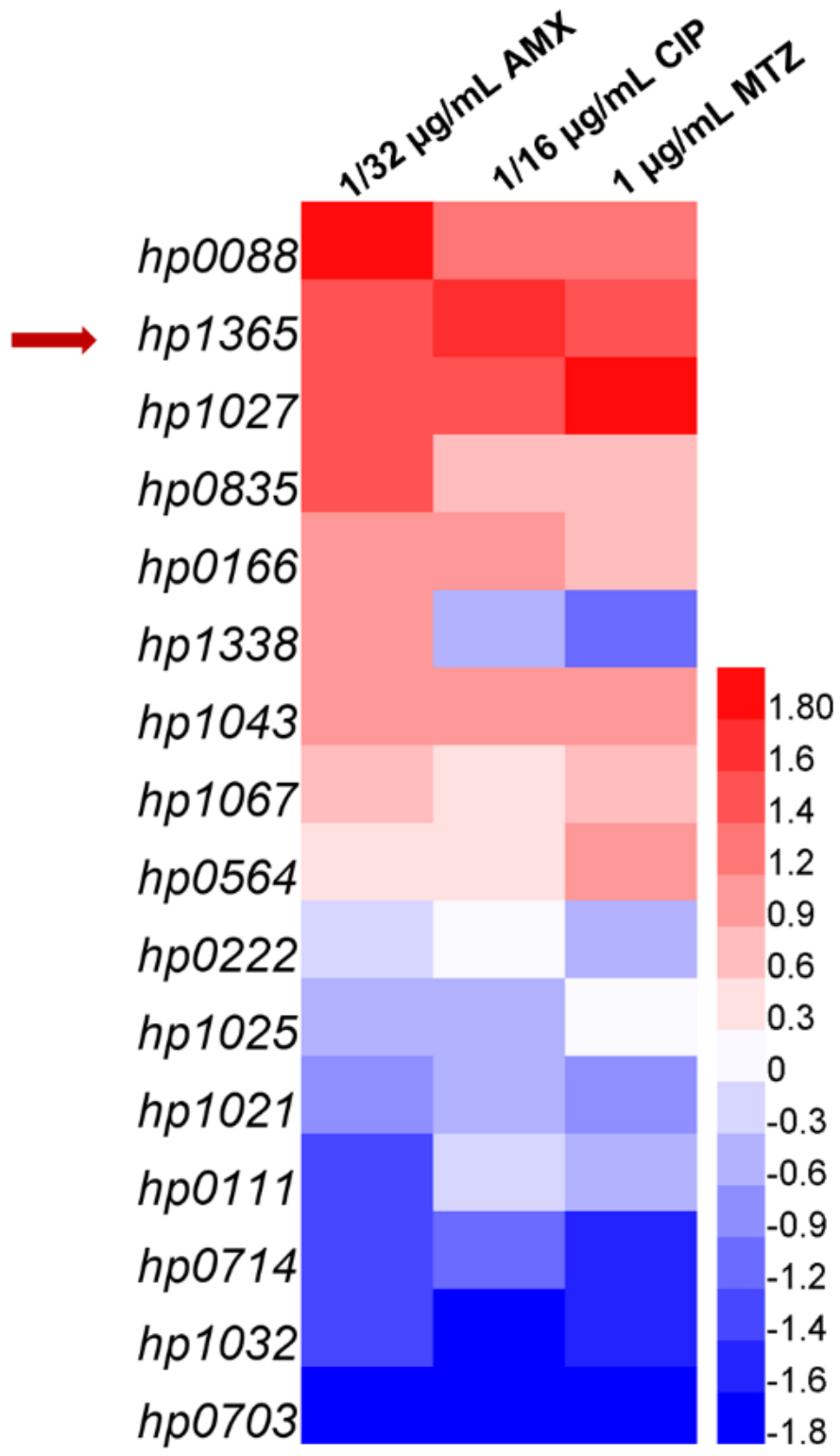

Fig S12 Transcriptomic expression profiling of regulatory genes in *H. pylori* WT biofilm cells induced by sub-inhibitory antibiotics. The heatmap displays differentially expressed genes in biofilm cells treated with sub-inhibitory MTZ, AMX, or CIP compared with untreated planktonic WT cells. Red indicates upregulation; blue indicates downregulation. The arrow marks *hp1365*, which encodes CrdR, the response regulator of the CrdRS two-component system.

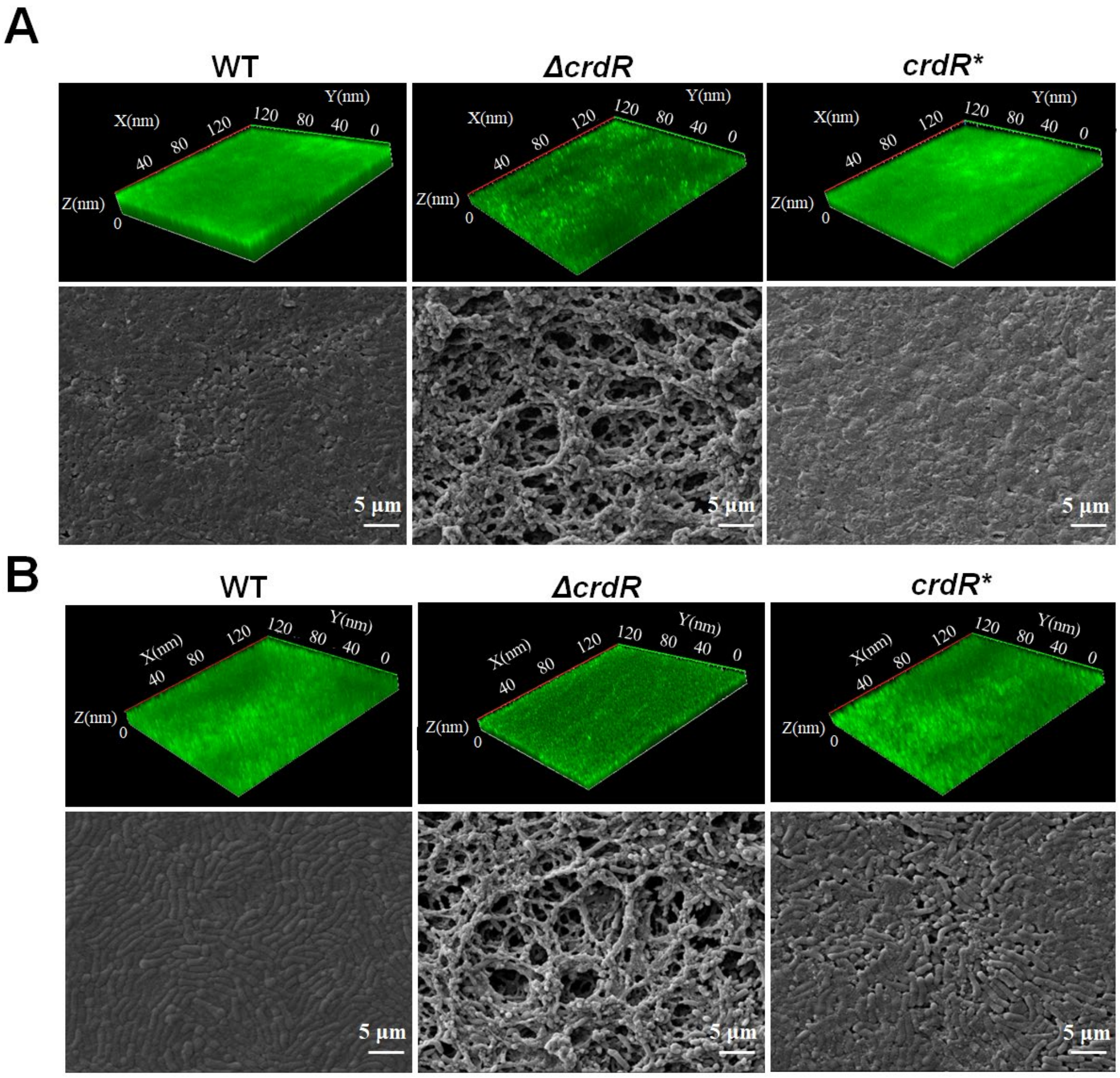

Fig S13 CrdR is required for sub-inhibitory AMX- and CIP-induced biofilm formation in *H. pylori*. WT, *ΔcrdR*, and *crdR** strains were cultured on medium containing AMX (1/32 μg/mL) or CIP (1/16 μg/mL) for 3 days to allow mature biofilm development. (A) Three-dimensional CLSM reconstructions (top) and SEM images (bottom; scale bar, 5 μm) of biofilms induced by AMX (1/32 μg/mL). (B) Three-dimensional CLSM reconstructions (top) and SEM images (bottom; scale bar, 5 μm) of biofilms induced by CIP (1/16 μg/mL). Data are representative of three independent experiments.

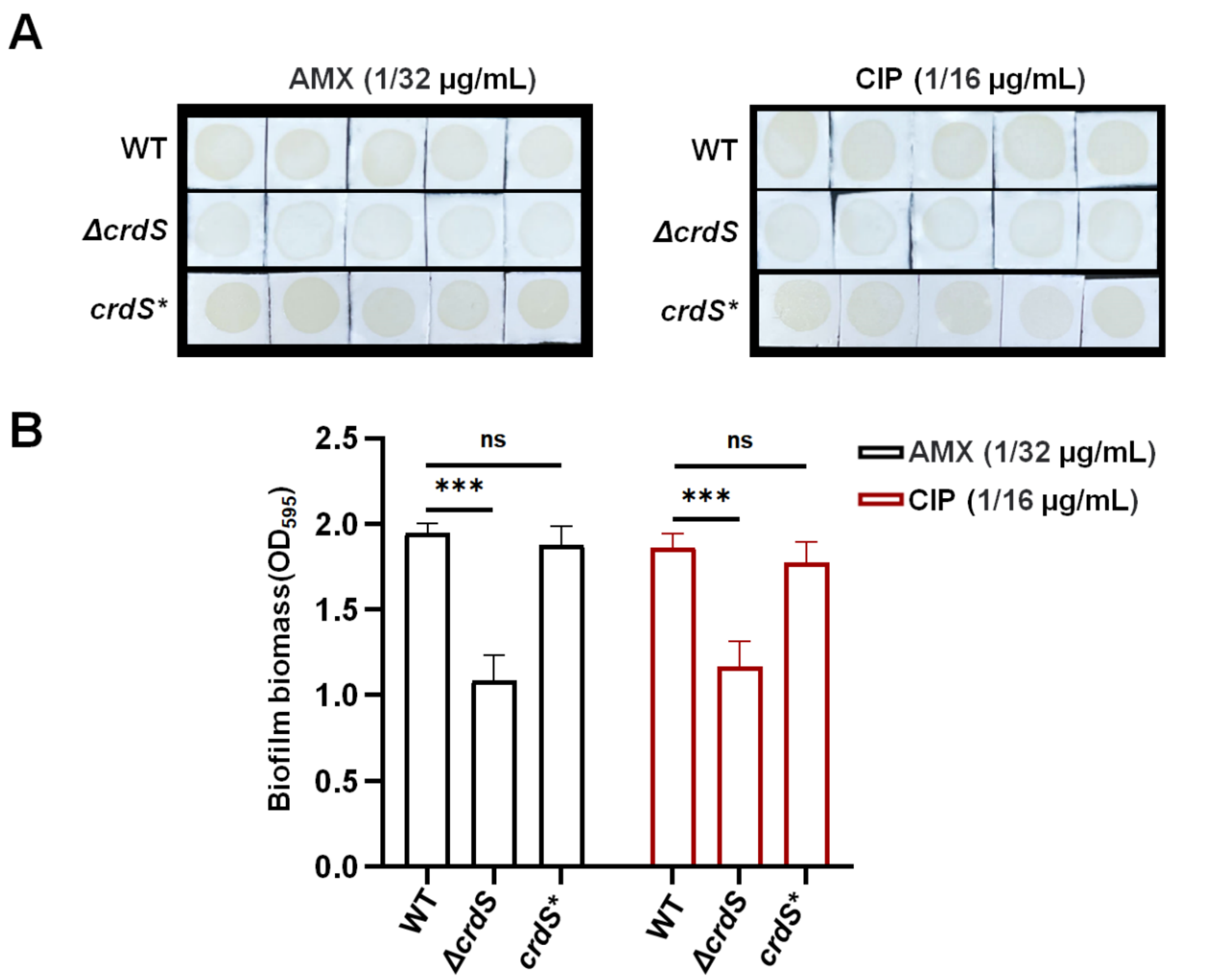

Fig S14 CrdS is required for sub-inhibitory AMX- and CIP-induced biofilm formation in *H. pylori*.(A) Representative images of biofilms formed on nitrocellulose membranes by WT, *ΔcrdS*, and *crdS** strains under AMX (1/32 μg/mL) or CIP (1/16 μg/mL) induction. (B) Biofilm biomass quantified by crystal violet staining (OD_{595}). Data are presented as mean ± SD from three independent experiments. Statistical significance: ns, not significant; ***, $P < 0.001$.

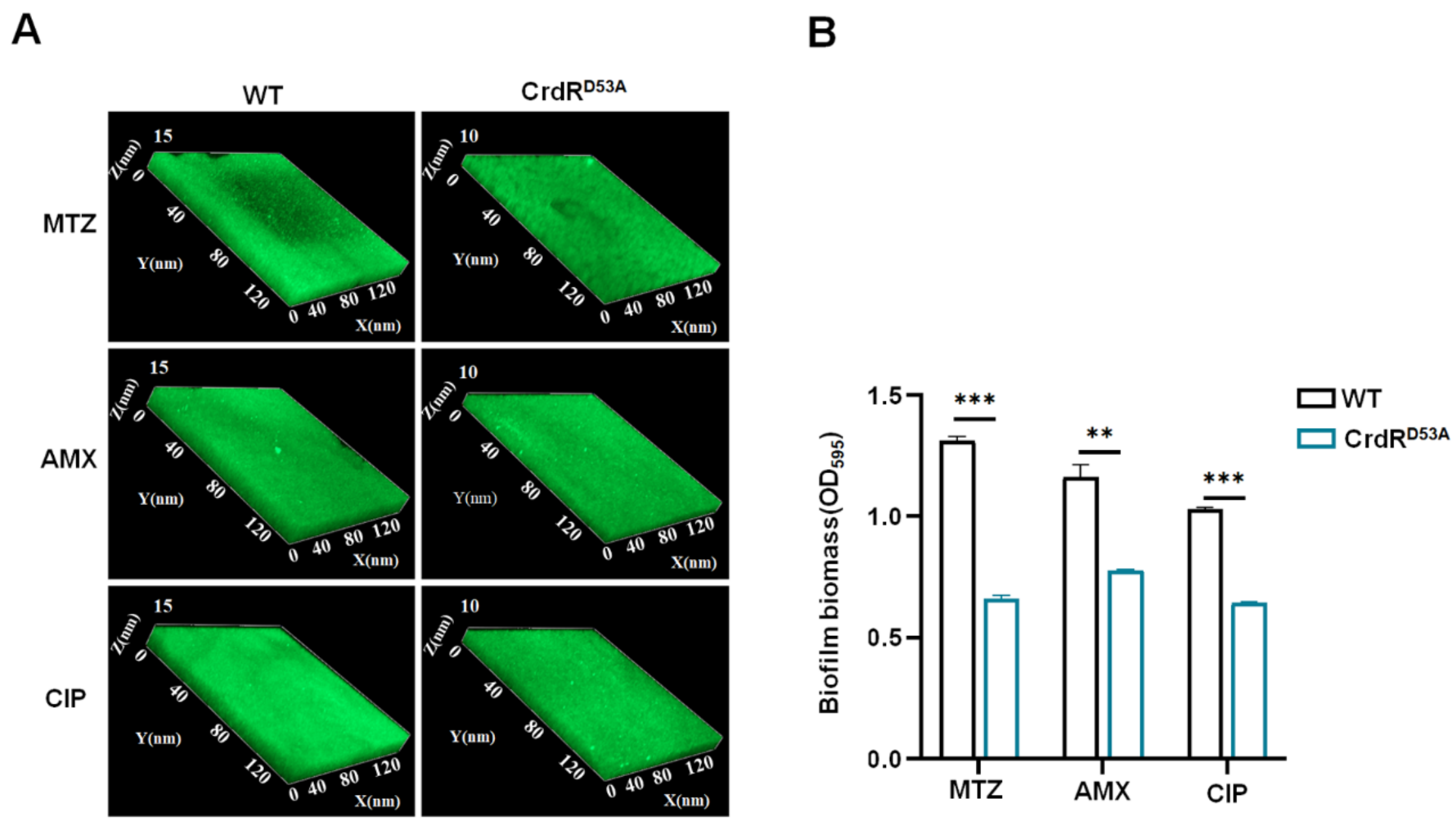

Fig S15 The CrdR^{D53A} phosphorylation site mutation impairs sub-inhibitory antibiotic-induced biofilm formation. (A) Three-dimensional CLSM reconstructions comparing biofilm thickness (Z-axis) between WT and CrdR^{D53A} mutant strains under induction by MTZ (1 µg/mL), AMX (1/32 µg/mL), and CIP (1/16 µg/mL). (B) Biofilm biomass quantified by crystal violet staining (OD_{595}). Data are presented as mean ± SD from three independent experiments. Statistical significance: **, $P < 0.01$; ***, $P < 0.001$.

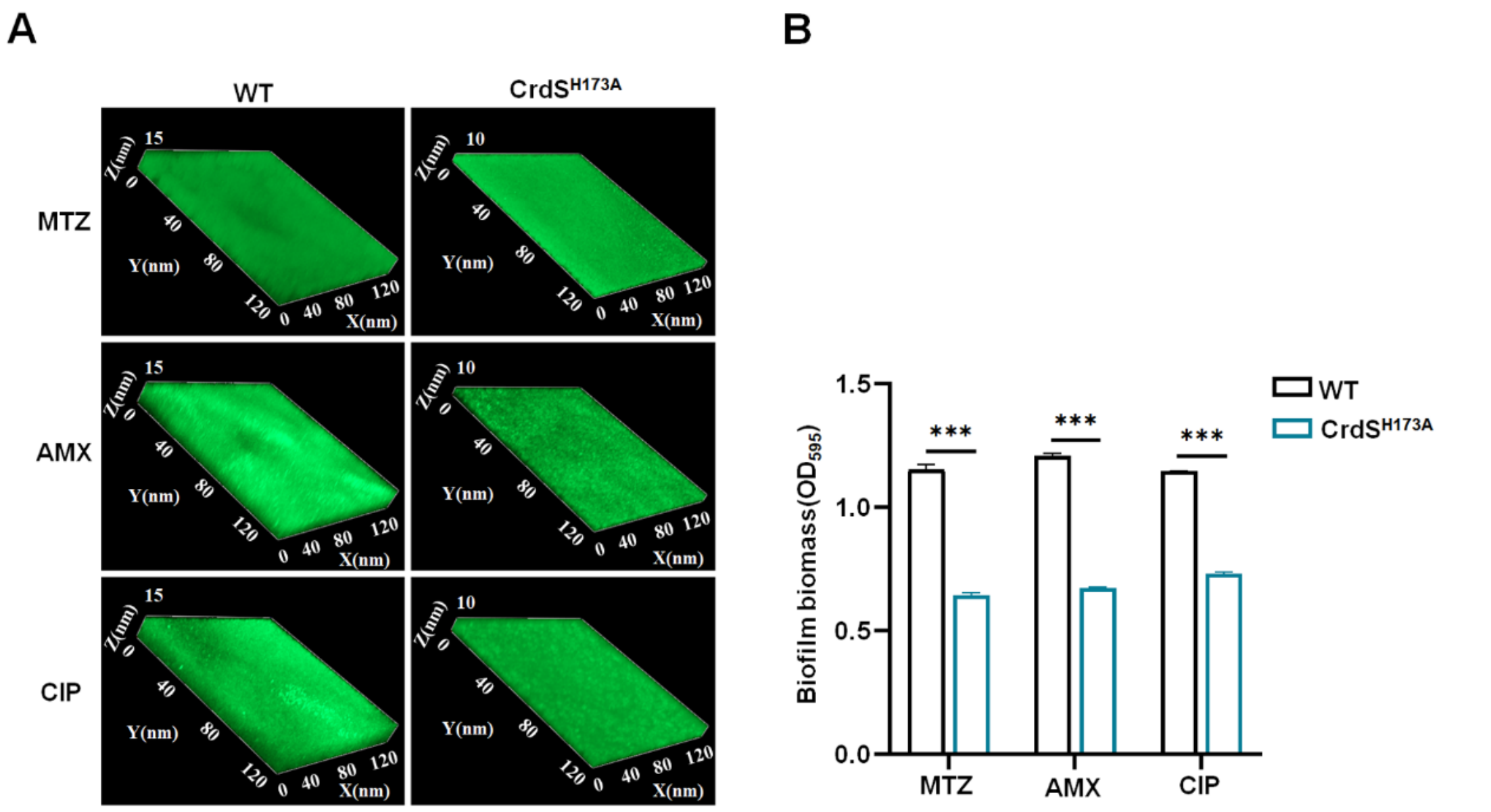

Fig S16 The CrdS^{H173A} phosphorylation site mutation impairs sub-inhibitory antibiotic-induced biofilm formation. (A) Three-dimensional CLSM reconstructions comparing biofilm thickness (Z-axis) between WT and CrdS^{H173A} mutant strains under induction by MTZ (1 µg/mL), AMX (1/32 µg/mL), and CIP (1/16 µg/mL). (B) Biofilm biomass quantified by crystal violet staining (OD_{595}). Data are presented as mean ± SD from three independent experiments. Statistical significance: **, $P < 0.01$; ***, $P < 0.001$.

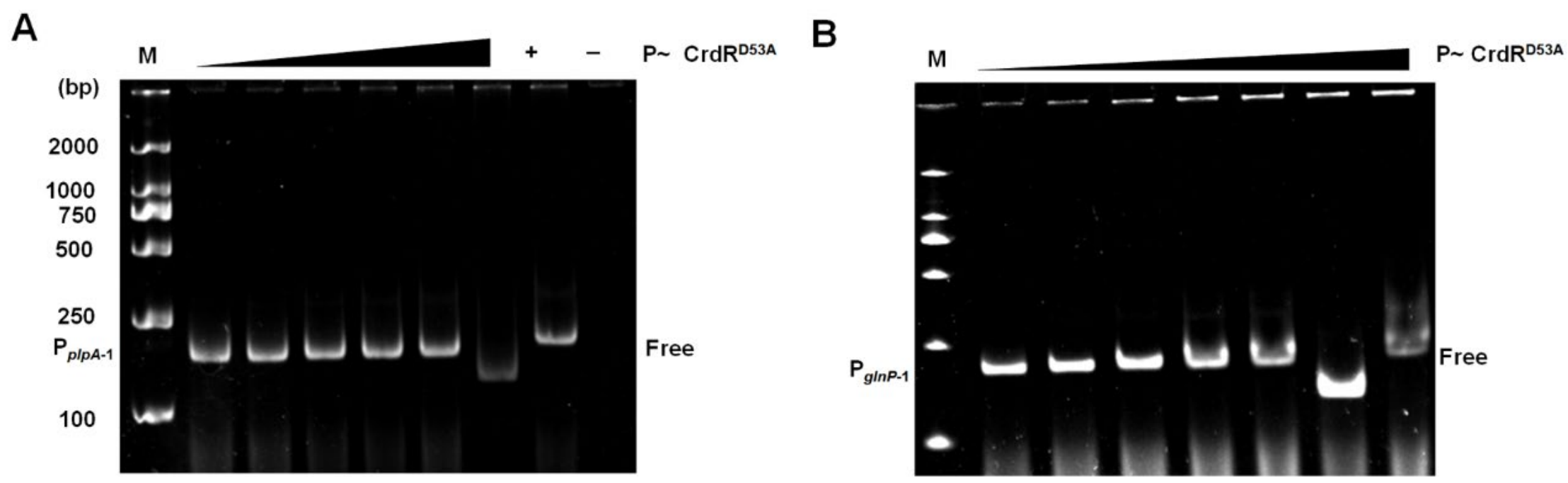

Fig S17 The CrdR^{D53A} mutant fails to bind the *plpA* and *glnP* promoter regions *in vitro*. (A) Phosphorylated CrdR^{D53A} does not form a shifted complex with probe $P_{plpA\text{-}1}$ containing dual AC-rich motifs. (B) Phosphorylated CrdR^{D53A} does not form a shifted complex with probe $P_{glnP\text{-}1}$ containing dual AC-rich motifs. Bound, DNA–protein complex; Free probe, unbound DNA probe. CrdR^{D53A} protein concentrations in lanes 1 to 5: 0, 3, 6, 9, 11.5, 11.5, and 11.5 µM (following phosphorylation treatment). Lane 6,

random sequence probe (negative control); lane 7, *crdA* promoter probe known to bind CrdR (positive control).

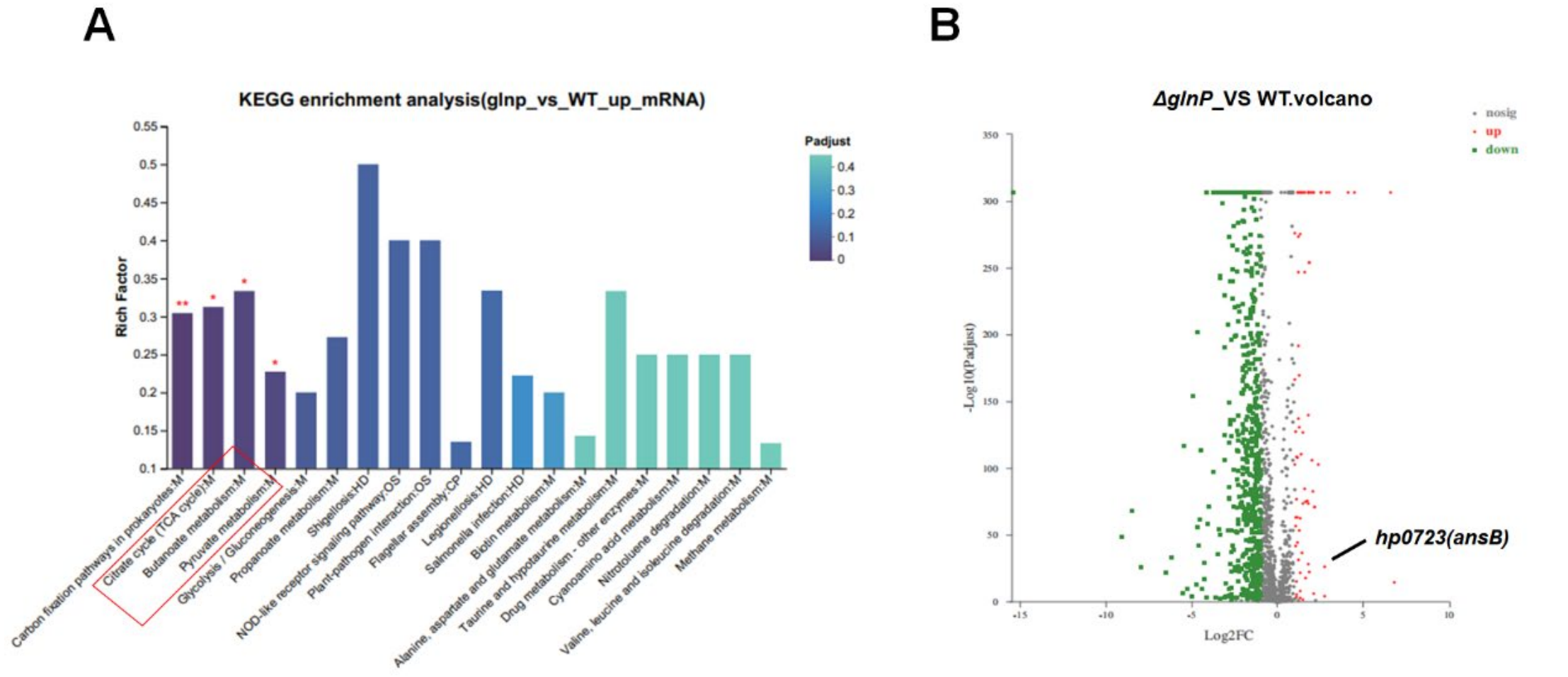

Fig S18 Transcriptomic analysis of *ΔglnP* mutant biofilms under sub-inhibitory MTZ stress. (A) KEGG pathway enrichment analysis of upregulated differentially expressed genes in *ΔglnP* versus WT biofilms. The x-axis represents enriched pathway names, the y-axis represents Rich Factor, and color indicates Padj value. Asterisks denote significantly enriched metabolic pathways, primarily including the tricarboxylic acid (TCA) cycle and amino acid metabolism pathways. (B) Volcano plot of differentially expressed genes in *ΔglnP* versus WT biofilms. The x-axis represents $\log_2$(fold change), and the y-axis represents $-\log_{10}$(Padj). Red, upregulated genes; green, downregulated genes; gray, non-differentially expressed genes. The arrow denotes *ansB* (*hp0723*), a metabolic node gene connecting the TCA cycle and amino acid metabolism pathways, which was significantly upregulated in the *ΔglnP* mutant.

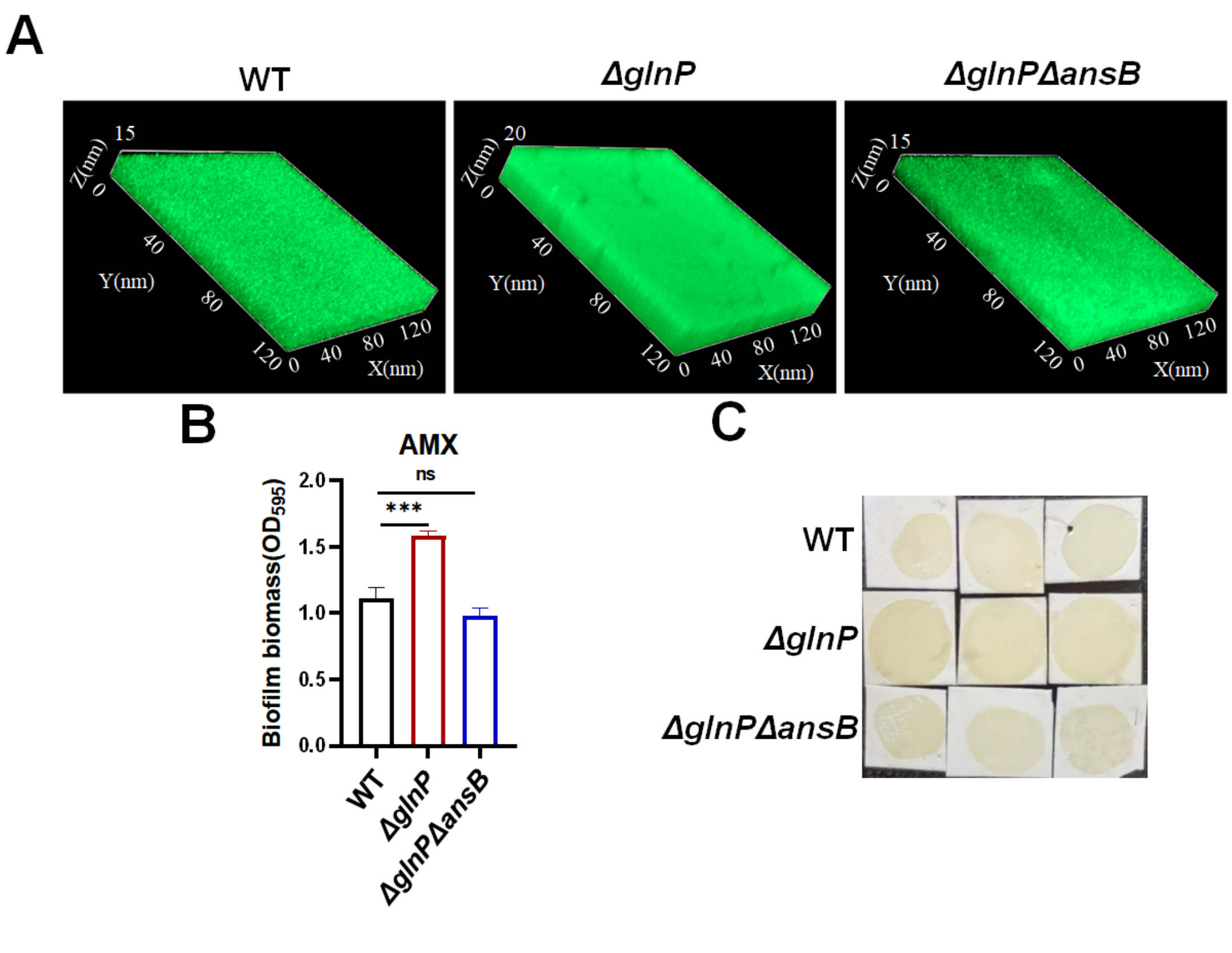

Fig S19 Deletion of *ansB* reverses the excessive biofilm formation phenotype of the *ΔglnP* mutant under sub-inhibitory AMX induction. (A) Three-dimensional CLSM reconstructions showing biofilm thickness (Z-axis) differences among WT, *ΔglnP*, and *ΔglnPΔansB* strains induced by AMX (1/32 μg/mL). (B) Biofilm biomass quantified by crystal violet staining (OD_{595}). (C) Representative macroscopic images of biofilms formed by each strain. Data are presented as mean ± SD from three independent experiments. Statistical significance: ns, not significant; ***, $P < 0.001$.

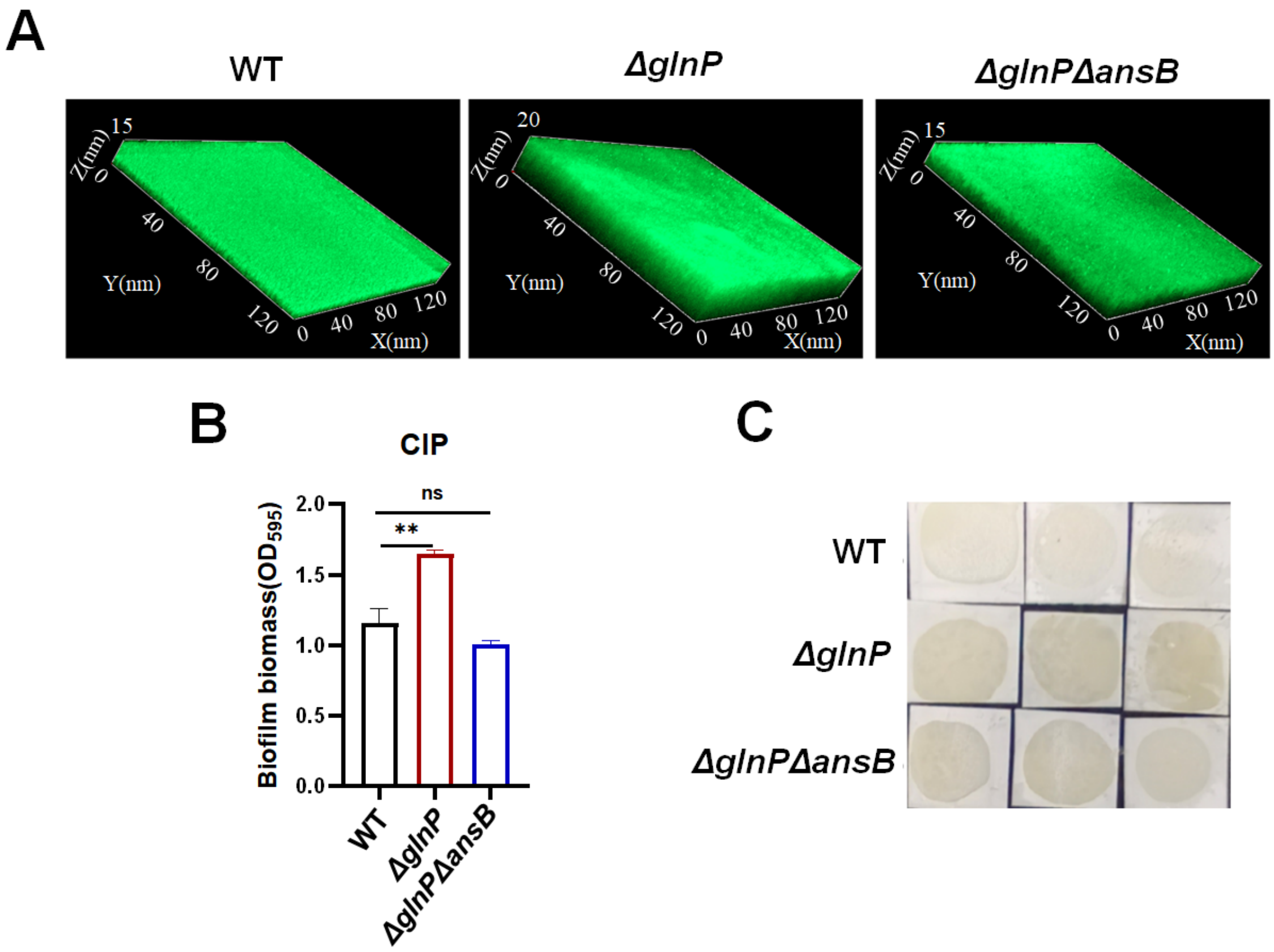

Fig S20 Deletion of *ansB* reverses the excessive biofilm formation phenotype of the *ΔglnP* mutant under sub-inhibitory CIP induction. (A) Three-dimensional CLSM reconstructions showing biofilm thickness (Z-axis) differences among WT, *ΔglnP*, and *ΔglnPΔansB* strains induced by CIP (1/16 μg/mL). (B) Biofilm biomass quantified by crystal violet staining (OD_{595}). (C) Representative macroscopic images of biofilms formed by each strain. Data are presented as mean ± SD from three independent experiments. Statistical significance: ns, not significant; **, $P < 0.01$.

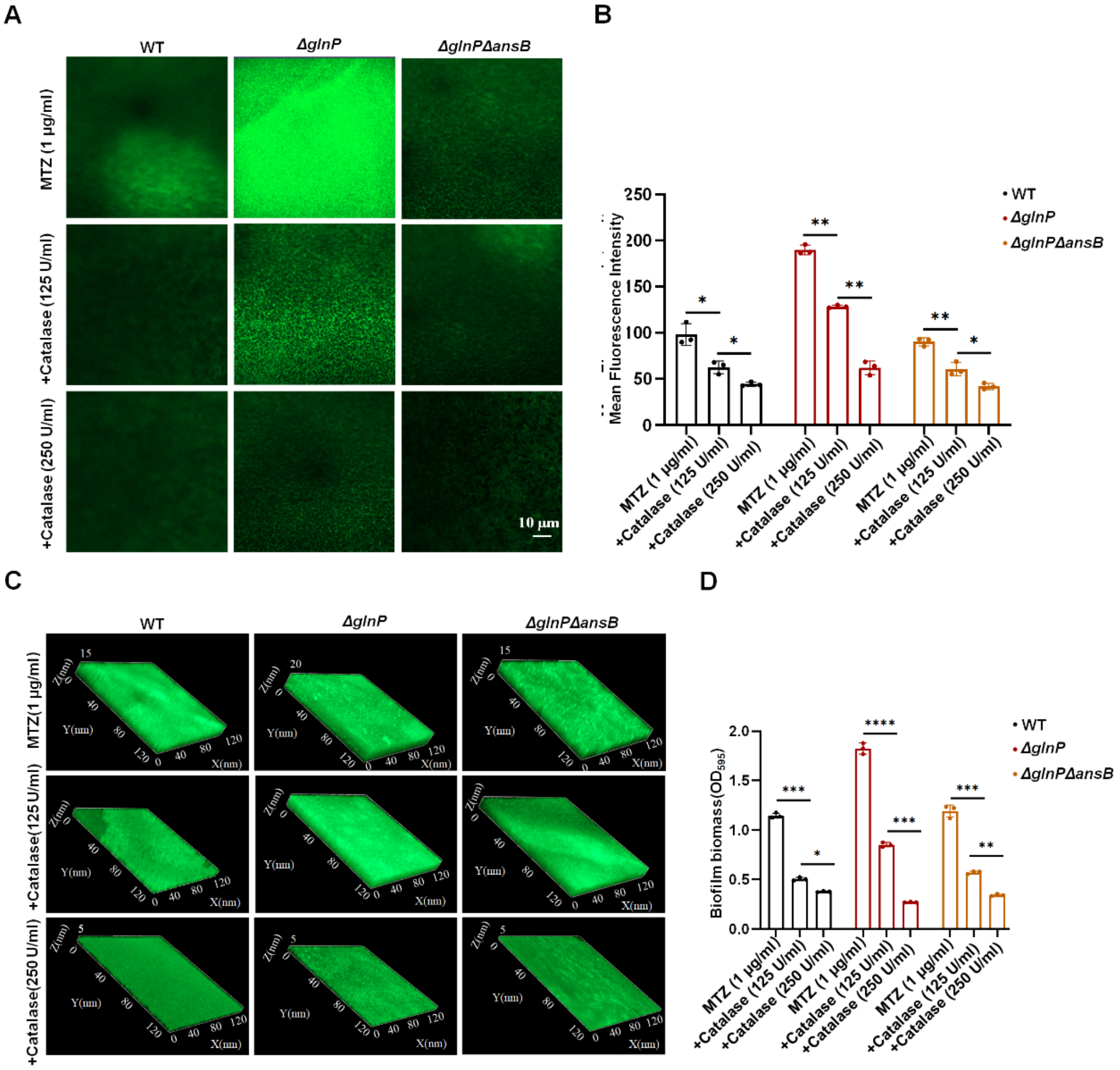

Fig S21 Catalase treatment inhibits MTZ-induced ROS accumulation and biofilm formation. (A) Intracellular ROS levels in biofilm cells of WT, *ΔglnP*, and *ΔglnPΔansB* strains under MTZ (1 µg/mL) treatment alone or in combination with catalase (125 and 250 U/mL), detected by CLSM with the DCFH-DA probe. scale bar, 5 µm. (B) Quantitative analysis of ROS fluorescence intensity. (C) Three-dimensional CLSM reconstructions showing biofilm thickness (Z-axis) for each condition. (D) Biofilm biomass quantified by crystal violet staining (OD_{595}). Data are presented as mean ± SD from three independent experiments. ns, not significant; Statistical significance:*, $P < 0.05$; **, $P < 0.01$; ***, $P < 0.001$; ****, $P < 0.0001$.

Table S1. Primers and their sequences used in this study. The suffixes "F" and "R" in each primer name denote forward and reverse primers, respectively.

Primers	Sequences (5'→3')
plpA-S-F	AACTGCAGAACAAGTGCATTTTGCATGG
plpA-S-R	GGAATTCATCATTTGGCACAGCAATCA
plpA-X-F	CGGGATCCCCTCAAAGACCCAAGCAATC
plpA-X-R	CCATCGATCGCAACCTTTTAAACGCTAA

*plpA**-F	CCATCGATATGAATGCATTCAAGCGTATT
*plpA**-R	AACTGCAGTTAAAAAGCCGGGATAATCG
glnP-S-F	AACTGCAGGCGTGGCTCTGCTATCATCT
glnP-S-R	CGGAATTCCCCACCACCGAAGTTTCTT
glnP-X-F	CGGGATCCCGCTTTTAGCATGGCTTTTT
glnP-X-R	CCATCGATTTTGGTCGCATGCATAAAGA
*glnP**-F	CCATCGATATGGCATTAGATTGGGATTTTATGT
*glnP**-R	AACTGCAGTTACCCTCTAATGTGTTGGAATTTTT
ansB-S-F	AACTGCAGTGTTTTGGCTCTGGCTCACT
ansB -S-R	CGGAATTCACGCCTTGAATACGGCTATCA
ansB -X-F	CGGGATCCATAATGCTGTGAGCGTAGCG
ansB -X-R	CCATCGATGGGGGAGCTTCATCAAATCT
crdR-S-F	AACTGCAGAAAAAGATTTTTTACTAGAAGACGATTAC
crdR-S-R	GGAATTCGGCTTTTTCAAATAATCGCTC
crdR-X-F	CGGGATCCGACGAATTGGAAGCACGCA
crdR-X-R	CCATCGATAGTGGGTTAAAGCGATAGCCA
*crdR**-F	CCATCGATATGCAAAAAAAGATTTTTTACTAGAAG
*crdR**-R	AACTGCAGTCATAGTGGGTTAAAGCGATAG
pET-*crdR*-F	CGGGATCCATGCAAAAAAAGATTTTTTACTAGAAG
pET-*crdR*-R	CCCTCGAGTAGTGGGTTAAAGCGATAGCCA
$P_{plpA\text{-}1}$-F	GAAAATTCCATTAAGCTTTAATCGG
$P_{plpA\text{-}1}$-R	AACTTTAAAACTAAGCGAAGAATC
$P_{plpA\text{-}2}$-F	TTGCAGAGTATCTCAAAGAAAATTC
$P_{plpA\text{-}2}$-R	AAAGAACTCTCTTGGTTAAAAG
$P_{plpA\text{-}3}$-F	CTTGGTTAAATCCTTTCTTTTTGATTC
$P_{plpA\text{-}3}$-R	GATAAAAAGATAAAACGATACGAAAAC
crdS-S-F	AACTGCAGAGGAGCGAGCGATTATTTGA
crdS-S-R	GGAATTCATGATCAATGCGGGTGATTT
crdS-X-F	CGGGATCCCCCATGAGCGCTTTAGTCTT
crdS-X-R	CCATCGATGGCGTTTTCTTCGTATGCTT
*crdS**-F	CCATCGATTTGGCTATCGCTTTAACCCACTATG
*crdS**-R	AACTGCAGTTATCCTTGAAATTGAACGCAAAAC
$CrdR^{D53A}$-S-F	AACTGCAGCTTGGCTCGCTCTATAGAAGAATTTGATC
$CrdR^{D53A}$ -S-R	GCACTTGCACTGCTAAAAGCAAGAGGT
$CrdR^{D53A}$ -X-F	ACCTCTTGCTTTTAGCAGTGCAAGTG
$CrdR^{D53A}$ -X-R	CGGAATTCTCATAGTGGGTTAAAGCGATAGCC
$CrdS^{H173A}$ -S-F	CGGGATCCGTGTTGATGCTAGTGATTAGCGTT
$CrdS^{H173A}$ -S-R	GGGGTGTTTAATTCAGCCGTGGTGTTTT

CrdS^{H173A} -X-F	AAAACACCACGGCTGAATTAAACACCCC
CrdS^{H173A} -X-R	CCATCGATAACCTAACCCTATACCATAACCC
PET- *crdR*^{D53A} -SF	AACTGCAGCTTGGCTCGCTCTATAGAAGAATTTGATC
PET- *crdR*^{D53A} -SR	GCACTTGCACTGCTAAAAGCAAGAGGT
PET- *crdR*^{D53A} -XF	ACCTCTTGCTTTTAGCAGTGCAAGTG
PET- *crdR*^{D53A} -XR	CCCTCGAGTAGTGGGTTAAAGCGATAGCC
$P_{glnp\text{-}1}$-F	TGTTGTTAATTTCAGCCAGTTTTTTT
$P_{glnp\text{-}1}$-R	AACGCTATACCTTAAAAATTAATGACG
$P_{glnp\text{-}2}$-F	GGGTGAATAAGAGCTGT
$P_{glnp\text{-}2}$-R	AACGCTATACCTTAAAAATTAATGACG
$P_{glnp\text{-}3}$-F	TGTTGTTAATTTCAGCCAGTTTTTTT
$P_{glnp\text{-}3}$-R	AACAGCTCTTATTCACCC
16s rRNA-F	GTGTGGGAGAGGTAGGTGGA
16s rRNA-R	GTTTAGGGCGTGGACTACCA
hp1564 rRNA-F	GGCTATCATAACAGGGAATTATG
hp1564 rRNA-R	TTCATCTTGCGCGTTATCC
Hp0298 rRNA-F	GCTAAACGCGTTTCGGA
Hp0298 rRNA-R	AACTTTAGACAAATGCGGCA
Hp1561 rRNA-F	GGCACTTCTACATAGCTCCC
Hp1561 rRNA-R	TTGAACGTGTGCAACGC
hp0889 rRNA-F	GGAGCGCTAAAATCACTCG
hp0889 rRNA-R	GAGTCCTTGAGCTTGGAAGAAT
hp0323 rRNA-F	GATCATTGATGATAAGATCGTGTTT
hp0323 rRNA-R	GTAATAGCTCTTGGCTTTGAGGA
hp1169 rRNA-F	CGCTTTGAGTTTGGGGTTTA
hp1169 rRNA-R	AAATCACATTCGCCCCTATG
hp1170 rRNA-F	TTCTCTCATTGGAGCGATTG
hp1170 rRNA-R	AACGCCATAAACCACAAAGC
